# Scientific assessment of the use of sugars as cigarette tobacco ingredients: A review of published and other publicly available studies

**DOI:** 10.3109/10408444.2011.650789

**Published:** 2012-01-20

**Authors:** Ewald Roemer, Matthias K Schorp, Jean-Jacques Piadé, Jeffrey I Seeman, Donald E Leyden, Hans-Juergen Haussmann

**Affiliations:** 1 Philip Morris International Management S.A., Operations Technical Services, Neuchâtel, Switzerland; 2 Philip Morris Products S.A., Philip Morris International Research & Development, Neuchâtel, Switzerland; 3 SaddlePoint Frontiers, Richmond, Virginia, USA; 4 Hardyville, Virginia, USA; 5 Toxicology Consultant, Roesrath, Germany

**Keywords:** Cigarette, tobacco, ingredient, sucrose, invert sugar, high fructose corn syrup, smoke, smoking behavior, nicotine exposure, risk

## Abstract

Sugars, such as sucrose or invert sugar, have been used as tobacco ingredients in American-blend cigarettes to replenish the sugars lost during curing of the Burley component of the blended tobacco in order to maintain a balanced flavor. Chemical-analytical studies of the mainstream smoke of research cigarettes with various sugar application levels revealed that most of the smoke constituents determined did not show any sugar-related changes in yields (per mg nicotine), while ten constituents were found to either increase (formaldehyde, acrolein, 2-butanone, isoprene, benzene, toluene, benzo[*k*]fluoranthene) or decrease (4-aminobiphenyl, N-nitrosodimethylamine, N-nitrosonornicotine) in a statistically significant manner with increasing sugar application levels. Such constituent yields were modeled into constituent uptake distributions using simulations of nicotine uptake distributions generated on the basis of published nicotine biomonitoring data, which were multiplied by the constituent/nicotine ratios determined in the current analysis. These simulations revealed extensive overlaps for the constituent uptake distributions with and without sugar application. Moreover, the differences in smoke composition did not lead to relevant changes in the activity in *in vitro* or *in vivo* assays. The potential impact of using sugars as tobacco ingredients was further assessed in an indirect manner by comparing published data from markets with predominantly American-blend or Virginia-type (no added sugars) cigarettes. No relevant difference was found between these markets for smoking prevalence, intensity, some markers of dependence, nicotine uptake, or mortality from smoking-related lung cancer and chronic obstructive pulmonary disease. In conclusion, thorough examination of the data available suggests that the use of sugars as ingredients in cigarette tobacco does not increase the inherent risk and harm of cigarette smoking.

## Contents

Abstract 244

1. Introduction 245
2. Methodological approach 246
  2.1 Guidance for toxicological assessment of cigarette ingredients 246
  2.2. Challenges for comparative testing of ingredients in a cigarette matrix 247
3. Fate of natural tobacco sugars and use of sugars as tobacco ingredients 248
4. Biological data related to transferred unchanged sugars 249
  4.1. Transfer of unchanged sugars to mainstream smoke 249
  4.2. Absorption, distribution, and metabolism of sucrose, fructose, and glucose after inhalation 250
  4.3. Physiological properties of sugars 250
  4.4. Toxicological properties of sugars 250
5. The effect of sugars on mainstream smoke composition 251
  5.1. Pyrolysis experiments with sugars 251
  5.2. Pooled quantitative chemical analyses of research cigarettes with and without sugars applied as tobacco ingredients 252
    5.2.1. Formaldehyde 254
    5.2.2. Acetaldehyde 256
    5.2.3. Acrolein and 2-butanone 256
    5.2.4. Volatile hydrocarbons 257
    5.2.5. N-Nitrosamines 257
    5.2.6. 4-Aminobiphenyl 257
  5.3. Additional constituents of mainstream smoke 257
  5.4. Summary, mainstream smoke chemistry 258
  5.5. Toxicological interpretation of changes in mainstream composition 258
6. Effects of using sugars as ingredients in toxicological assays 259
  6.1. *In vitro* cytotoxicity 259
  6.2. *In vitro* genotoxicity 260
  6.3. Sub chronic inhalation toxicity 260
  6.4. *In vivo* genotoxicity 263
  6.5. Carcinogenicity studies 264
  6.6. Summary, toxicological assays 264
7. Comparison of marketed American-blend and Virginia-type cigarettes 265
  7.1. Relative impact of sugar application on chemical composition of mainstream smoke in marketed American-blend and Virginia-type cigarettes 265
  7.2. Comparison of marketed American-blend and Virginia-type cigarettes in toxicological assays 266
  7.3. Simulated uptake in smokers of mainstream smoke constituents at maximum sugar application level and from marketed American-blend and Virginia-type cigarettes 267
  7.4. Comparison of American-blend and Virginia-type markets regarding smoking behavior 269
  7.5. Comparison of American-blend and Virginia-type markets regarding smoking-related disease risks 271
  7.6. Conclusion, comparison of marketed cigarettes 272
8. Limitations and uncertainties of this assessment 272
9. Overall conclusion 273

Declaration of interest 273
References 273

## 1. Introduction

Cigarette smoking has been established as addictive and the cause of various diseases, such as cancer, cardiovascular disease, and chronic obstructive pulmonary disease (COPD). However, little is known about the etiology of these diseases in terms of smoke composition and whether cigarette design characteristics or the use of ingredients might influence this composition. Because of the disease burden related to smoking, an improved understanding of such potential influences is of major toxicological and public health relevance. Such understanding may eventually support both the development of potentially less risky tobacco products (US Institute of Medicine, 2001) and of meaningful regulation.

Regulatory schemes for tobacco have been developing in various regions of the world over the past several years (World Health Organization Study Group on Tobacco Product Regulation, 2008), which also address the use of ingredients. To date, ingredients have been regulated in some countries by positive and negative lists (e.g. German Federal Ministry for Youth Family and Health, 1977; UK Department of Health, 2003), which would suggest a safe use of those listed as permitted. Nevertheless, this assumption has been questioned for certain ingredients (Talhout et al., 2006; Rabinoff et al., 2007), and in Canada, the use of many ingredients to tobacco including sugars was recently prohibited (Parliament of Canada, 2009). Apart from the regulatory status, investigations have been conducted in recent years in order to assess the potential risk (per cigarette) or harm (for a population), which might stem from the use of ingredients in addition to the inherent risk or harm from smoking tobacco manufactured into a cigarette (e.g. Carmines, 2002; Baker et al., 2004a). Data from individual toxicological studies performed by the tobacco industry have been submitted according to regulatory requirements (e.g. European Parliament and Council of the European Union, 2001, which is requesting all available toxicological study data). For a few ingredients, scientific assessments of the potential additional harm resulting of their use as cigarette ingredients were published (Talhout et al., 2006; Werley et al., 2006; Heck, 2010). In the current context, the term “harm” refers to a population-based risk and includes the potential that the use of ingredients may facilitate initiation, impede cessation, or increase the intensity of use, which in more recent guidance documents has been referred to as “indirect harm” (US Food and Drug Administration Center for Tobacco Products, 2011).

In the Western world, there are two major types of cigarettes that differ in their tobacco blending and ingredient content. Virginia-type cigarettes are mainly made from Virginia (or Bright) tobacco. In the blend preparation of American-blend cigarettes, a significant portion of the Virginia tobacco is replaced by air-cured tobaccos (usually Burley). Virginia tobacco is high in endogenous sugars, while cured Burley leaf is practically devoid of endogenous sugars due to catabolic breakdown during curing (Fisher, 1999; Leffingwell, 1999). A sensorially satisfactory adjustment of the total blend is achieved by applying a sugar casing to the tobacco blend of American-blend cigarettes, which in part replenishes the sugar content of the tobacco lost during Burley curing to a level that is generally below that found in Virginia-type cigarettes in order to rebalance the sugar/nitrogen ratio of cured Burley tobacco and to enhance the taste and smoke characteristics of the blend (Fisher, 1999). Thus, the use of sugars as tobacco ingredients is considered essential for an American-blend cigarette. The sugars used most widely as casing materials for tobacco are sucrose and invert sugar (the hydrolysis product of sucrose, mainly glucose and fructose). Both are approved for use in tobacco products as additives, flavorings, or flavoring agents in all countries that have a list of ingredients that are allowed to be added to tobacco. For example in Germany, food-grade sugars are generally allowed as additives to tobacco (German Federal Ministry for Youth Family and Health, 1977); in the UK, invert sugar and sucrose can each be added to a maximum of 10% of the weight of tobacco plus paper, with an aggregate limit of 15% of those additives typically used at higher application levels (List 1, UK Department of Health, 2003).

Because of the importance of sugars as tobacco ingredients in American-blend cigarettes, many experimental studies have been performed to date in order to better understand any potential toxicological impact of such use. This review evaluates the available information from published or non-published studies based on searches in Medline and the documents of different cigarette manufacturers gathered in the Legacy Tobacco Document Library (http://legacy.library.ucsf.edu/). The information discussed extends that in a previous review on the impact of using sugars as ingredients (Talhout et al., 2006), e.g. by including *in vitro* and *in vivo* studies on the impact of sugars added as tobacco ingredients in research cigarettes or by evaluating their potential impact in commercial cigarettes, such as on smoking behavior. The overall objective for this assessment is to evaluate on a weight-of-evidence basis whether the use of sugars as tobacco ingredients increases the known risk and harm, which is inherent to tobacco smoking, or whether new risks are generated. After a short description of the methodological approach used for this assessment, this review includes information on

- technical data on the use of sugars as ingredients in cigarette tobacco,
- the fate of sugars during the manufacturing process of cigarettes,
- the unchanged transfer of sugars to mainstream smoke and their potential direct physiological and toxicological effects,
- the degradation during pyrolysis or during burning in the cigarette matrix,
- the analysis of the chemical composition of the mainstream smoke of research cigarettes with or without the application of sugars to tobacco,
- the toxicity of the smoke of such research cigarettes in *in vitro* and *in vivo* assays, and,
- a comparison of smoke composition, smoking behavior and exposure, as well as disease risk data from predominantly American-blend and Virginia-type cigarette markets to provide an indication of potential differences in the harm stemming from these major types of commercial cigarettes with principle differences in their content of ingredients, such as sugars.

## 2. Methodological approach

### 2.1 Guidance for toxicological assessment of cigarette ingredients

A generally accepted regulatory guidance for the assessment of existing or new cigarette ingredients is not available. However, various individual guidance documents and recommendations were published over the last several years (Dempsey et al., 2011). The US Institute of Medicine described some regulatory principles, which include the use of appropriate toxicological testing methods “with the objective of identifying those ingredients that add no significant toxicity to tobacco products and therefore can be considered safe in the context of this use” (US Institute of Medicine, 2001). In the UK, a voluntary agreement suggested that apart from studies on transfer and pyrolytic degradation of an ingredient, a toxicological assessment of new ingredients should include *in vitro* genotoxicity and subchronic inhalation studies, preferentially in the cigarette matrix (Tobacco Manufacturers Association & UK Department of Health, 1997). In Germany, a guidance document was published suggesting the comparative and quantitative testing of ingredients in research cigarettes with and without the ingredient, which may include a series of *in vitro* tests that may be supplemented by chemical analyses and *in vivo* testing (Deutsches Institut für Normung - Working Group Toxicology of Additives, 2004; Hahn and Schaub, 2010). The most encompassing recommendation was published by the Life Science Research Office, which evaluated the feasibility of testing and laid out scientific criteria for the evaluation of ingredients added to tobacco (Life Sciences Research Office, 2004a,b). The above-mentioned guidance documents assume the feasibility and relevance of a toxicological evaluation of ingredients as a design principle, i.e. the evaluation of an ingredient for use in cigarette tobacco in general without the need to be repeated for each particular brand in which this ingredient is used. However, the World Health Organization study group on Tobacco Regulation concluded that the existing science base would currently not be sufficient to allow regulation based on design characteristics (Burns et al., 2008; World Health Organization Study Group on Tobacco Product Regulation, 2008). Rather, this group felt that a more robust tool might be the regulation of the smoke composition of commercial products (brands).

This review attempts to consider both of these general positions with the intention of considering all available information: sucrose and invert sugar were evaluated as neat compounds as well as in research cigarettes with or without sugars applied as single ingredient or in combinations of ingredients. In addition, commercial American-blend and Virginia-type cigarettes were compared, which apart from some other factors significantly differ by the use of sugars and other ingredients.

### 2.2. Challenges for comparative testing of ingredients in a cigarette matrix

Ingredient mixtures differ between brands and even within a given brand because of country-specific preferences. Because of practical reasons and in line with the practice in other industries (e.g. for food additives: European Commission Health and Consumer Protection Directorate-General, 2001), ingredients have usually been evaluated as design principles rather than repeatedly for each product. For principle-based testing, ingredients may be applied to research cigarettes, either individually or in combination, in a manufacturing process which corresponds to that of commercial cigarettes. Several levels of ingredient application are required to allow the determination of potential “dose-response” relationships. The use of a combination of ingredients includes the investigation of any potential interactions among the ingredients, while the use of single ingredients allows greater exaggeration of the application levels of the particular ingredient beyond use levels found in commercial cigarettes. Such exaggeration may improve the potential to detect any adverse effects associated with the application of this ingredient, although it has its limits for the testing of ingredients at high unusual application levels if significant changes in the burning characteristics of the cigarette would occur which cannot be back-extrapolated to the ingredient levels in actual products. Both types of studies using single or combined ingredients were included in this review.

Cigarettes may be smoked in many ways by varying puffing parameters, such as puff duration or volume, which will change the composition of the resulting smoke in quantitative terms. For toxicological testing purposes, fixed machine-smoking conditions need to be maintained in comparative assessments to ensure comparative smoke generation conditions for testing (Burns et al., 2008; World Health Organization Study Group on Tobacco Product Regulation, 2008). The fixed smoking conditions are not considered to be representative of a human smoker or a group of smokers. While very intense machine-smoking conditions have been recommended for future tests for maximum yield (Burns et al., 2008), this may be disadvantageous for the discriminatory power of chemical-analytical and toxicological tests (Roemer and Carchman, 2011). Until now, most previous tests were conducted using smoking conditions in line with FTC (US Federal Trade Commission, 1967) or ISO (International Organization for Standardization, 2000) conditions using puffs of 35 ml taken over 2 s, once per minute. The appropriateness of this protocol versus a more intense smoking condition was evaluated in the current assessment in terms of potential ingredient-related changes in smoke composition.

Various approaches to compare the results of a series of research cigarettes or cigarette brands were used in the past, depending on the objectives of the particular studies. For instance, precursor-constituent relationships may best be evaluated on the basis of total particulate matter (TPM), tar (TPM minus water and nicotine), or carbon monoxide yields. For chemical-analytical investigations and related risk extrapolations, nicotine has been suggested as the reference smoke constituent for comparative evaluations (Burns et al., 2008; World Health Organization Study Group on Tobacco Product Regulation, 2008). For the current assessment, nicotine was chosen as the basis for comparison in all chemical-analytical studies. It should be noted that the research cigarettes in this type of comparative studies were generally made to the same cigarette weight by replacing tobacco with the ingredients to be tested. This can be considered worst case for the evaluation of potential effects of ingredients, because the cigarette nicotine content and thus the smoke yield of nicotine as the basis of the comparison automatically decreases with increasing ingredient applications levels. This is particularly relevant for the exaggerated application levels in some research cigarettes. For the comparison of the results of *in vitro* and *in vivo* studies, TPM was used as the basis of comparison in the current assessment, because TPM was the lead parameter for dosing in these studies. Besides, as for nicotine, using TPM as a basis of comparison rather than comparing on a per cigarette basis offers a comparison of the quality rather than the quantity of smoke used. As most of the presented *in vitro* and *in vivo* studies were carried out with experimental or even standard reference cigarettes of quite similar design, a relatively constant TPM/nicotine ratio can be assumed for data transformation.

As there is no authoritative recommendation for the toxicological hazard assessment of cigarette ingredients, toxicological test systems available for other regulatory environments, such as for pharmaceutical (e.g. International Conference on Harmonisation, 1997) or chemical (e.g. Organization for Economic Co-operation and Development, 2009) compounds, had to be selected and adapted to a comparative testing paradigm for cigarette ingredients. In most of the assays selected, cigarette smoke is inherently active, which requires testing protocols suitable to achieve a discriminatory power that is needed to detect potential incremental changes in activity by modifications in the research cigarettes, such as increasing levels of ingredients. In addition to biological testing and in view of the complexity of the smoke aerosol compared to, e.g., a pharmaceutical compound, rather comprehensive chemical analyses of potential changes in smoke composition by the use of ingredients have been conducted. Thus, assessing the toxicity of cigarette smoke requires both chemical analyses and biological assays for the intended comparative hazard assessment. Chemical analyses relatively precisely determine increases and decreases in mainstream smoke yields of defined constituents. However, predicting the impact of these increases and decreases on the overall toxicity of cigarette smoke is a major challenge. Biological assays are rather unspecific regarding the constituents that cause a response and include the assessment of potential biological interactions among constituents. Therefore, chemical and biological assays complement one another. In the current assessment, the results of chemical, *in vitro*, and *in vivo* studies were pooled for each endpoint investigated attempting to derive quantitative results across studies dependent on the level of sugars applied.

Using the assumption of proportional constituent/ nicotine uptake ratios, the hypothetical uptake of smoke constituents from research cigarettes with or without applied sugars in a given population was modeled on the basis of known uptake distributions for nicotine. This should allow an evaluation of the potential impact of a change in the uptake of a given smoke constituent due to changes in smoke composition triggered by the use of sugars as ingredients *vis-à-vis* the broad variability of uptake of this constituent in a population of smokers.

Exposure studies to determine whether the use of ingredients may change smoking behavior have not been performed, except for menthol (reviewed by Heck, 2010). Approaches for controlled clinical studies to address the potential impact of flavor ingredients on exposure were suggested (Life Sciences Research Office, 2004b), but have not been validated for general use to our knowledge. Instead, the current analysis includes a comparison of published data on smoking behavior parameters, such as smoking prevalence or nicotine uptake, in major markets of American-blend or Virginia-type cigarettes, which differ, among other factors, in their sugar content.

For a complex mixture that causes complex pathogeneses, there is no unique and simple approach to combine all information available for a final conclusion. Rather, various layers of evaluation that weigh the respective evidence need to be combined in a tiered approach.

## 3. Fate of natural tobacco sugars and use of sugars as tobacco ingredients

Since tobacco is a natural plant product, it contains high levels of carbohydrates, and sugars in particular (Fisher, 1999; Leffingwell, 1999). Green tobacco leaves require a drying step (curing) before use as a cigarette filler, which critically influences the sugar content of the end product. The first curing step common for all tobacco types is the “yellowing stage,” which is the color change that results from chlorophyll degradation that starts as soon as the green leaves start to dry. Enzymatic hydrolysis of starch into sugars begins in the early stages of curing, and proteins begin to fragment, releasing amino acids. After the yellowing phase, different curing conditions are used, depending on the variety of tobacco. Air-curing of Burley tobacco, which is grown using relatively high nitrogen fertilization and thus rich in nitrate, proteins, and amino acids, is performed by naturally drying the leaves at ambient temperature. This is a slow process that can last up to ten weeks. During this process, enzyme systems in the leaf remain active and sugars are catabolically consumed. As a result, the cured Burley leaves have very low (0.2%, Leffingwell, 1999) to not detectable (Fisher, 1999) sugar contents. In contrast, during flue-curing of Virginia tobacco used for Virginia-type cigarettes, the yellowing is followed by a relatively short (about 4 days) drying stage under controlled humidity and at elevated temperatures, which stops enzymatic processes, such as sugar catabolism, due to desiccative dehydration but allows the degradation of starch to even increase the natural sugar content in the cured leaf. Residual sugar levels of 8–30% have been reported in flue-cured tobacco (Fisher, 1999). Virginia tobacco is also low in nitrogen-containing compounds, because of cultivar selection and the limited requirement for nitrogen fertilization. The sun-curing of Oriental tobacco does not involve controlling air temperature or humidity, but it is fairly similar to flue-curing in that the enzymatic processes are much more rapidly stopped than in air-curing. The natural sugar content of Oriental tobacco is intermediate (10–20%).

The core blend components in American-blend filler consist of approximately 50% Virginia, 30% Burley, and 20% Oriental (assuming proportional contributions from expanded and reconstituted materials to either tobacco type) according to the composition of the 2R4F reference research cigarette provided by the University of Kentucky (Chambers, 2003), which was designed to be representative for the American-blend markets (Chepiga et al., 2000; Roemer et al., 2004; Patskan et al., 2008). These tobaccos are of different grades, origins and crop-years. Virginia-type cigarettes mainly consist of Virginia tobacco (including expanded and reconstituted materials) with little or no Oriental tobacco. Thus, in American-blend cigarettes, a substantial part of the Virginia tobacco is replaced by Burley (and Oriental), which accordingly reduces the natural sugar content of the blend. This loss is partly replenished by applying sugars as tobacco ingredients. The application of sugar-containing casings is thus a key part of the manufacturing of the American-blend filler, in contrast to Virginia-type cigarettes. Generally, not more than 5% (w/w) of sugar is applied to American-blend cigarettes. This results in a similar or lower total sugar content in American-Blend compared to Virginia-type cigarettes. In the published literature, data on the actual sugar content in marketed cigarette tobacco can hardly be found, but the above theoretical calculation was confirmed by a comparison of American-blend and Virginia-type cigarettes marketed in the UK reporting 11 and 16% total sugar content and 9 and 13% reducing sugar content, respectively (Woertz, 1983). Average reducing sugar contents of 6.6 and 9.6% were reported for US American-blend and Australian Virginia-type cigarette brands, respectively (Scollo and Winstanley, 2008).

**Figure 1:**
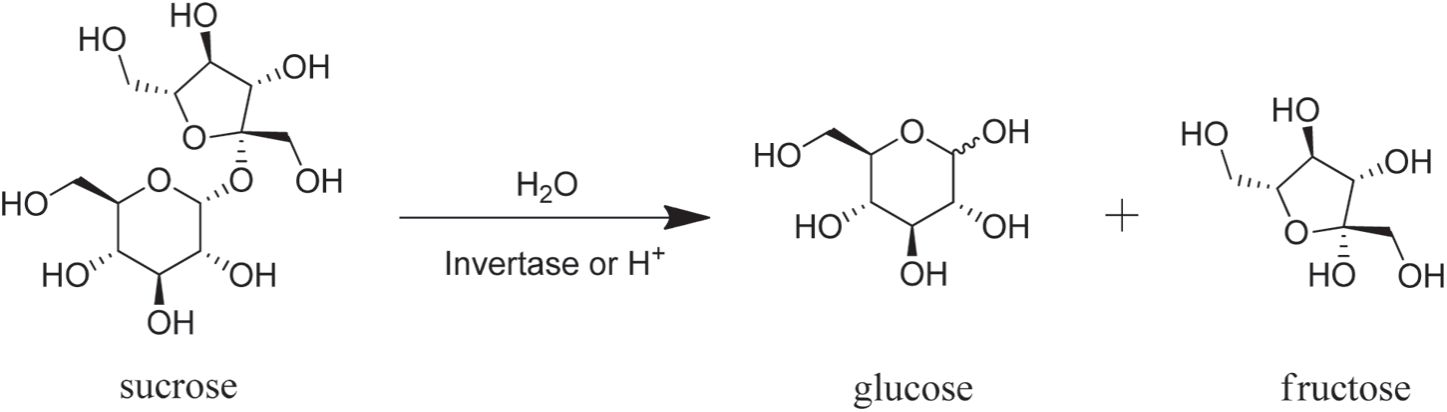
Chemical structures demonstrating the inversion of sucrose to glucose and fructose.

The sugars most widely used as cigarette tobacco ingredients are sucrose and invert sugar. Sucrose (table sugar, saccharose), one of the most widely distributed disaccha-rides, is obtained commercially from sugarcane and beets. It is used in the granulated form, crystallized from the highest purity sugar liquor and, thus, is the highest-purity crystalline sugar product. Conversion (inversion) of the disaccharide sucrose into its two monosaccharide components yields glucose and fructose. Invert sugar is the water-based mixture resulting from the almost complete inversion of sucrose (Figure 1). Sugars suitable for human consumption are used as cigarette tobacco ingredients (generally recognized as safe (GRAS) for human oral consumption: US Food and Drug Administration, 2000a,b).

The only processing steps in the cigarette factory during which sugars may further react are flue-cured tobacco expansion, i.e. increasing the volume of the tobacco leaves, and Burley tobacco drying after casing, i.e. after the application of water soluble cigarette ingredients. The expansion process is performed at a very high temperature (ca. 350°C) under an inert atmosphere over a very short period of time (1–2 s). A certain percentage of the flue-cured tobacco sugar contents is consumed during the process, depending on the conditions (Green et al., 2007). The main products formed in the process appear to be sugar polymers and degradation products. Most volatile and semi-volatile products that would be produced will be lost in the process depending on their volatility. Burley tobacco drying after casing is typically performed over 5–6 min, and the tobacco reaches a temperature of about 75°C at the end of the drying process (Abdallah, 2004). In fact, except for the much shorter heating time, Burley drying conditions are very similar to flue-curing conditions. Under these drying conditions, no change of the total sugars levels in the tobacco could be detected. The largest effect of the thermal treatment, besides water evaporation, is the release of ammonia. An increase in the levels of reaction products of sugar-ammonia reaction products was also reported (Moldoveanu et al., 2011).

Carbohydrates and nitrogen compounds in tobacco are important sources of flavor compounds in the smoke of tobacco blends (Noguchi et al., 1971; Dickerson et al., 1976; Leffingwell, 1976). Maillard reactions involving amino acids or ammonia and sugars are ubiquitous during the curing, storing, processing, and particularly during smoking of tobacco. Maillard reaction products are very diverse in chemical structure and flavoring potential. Many of them are heterocyclic compounds, a most important class being pyrazines. Caramelization refers to non-enzymatic browning reactions in which, unlike in the Maillard reactions, sugars do not react with amines. These reactions proceed through a complex series of oxidation, dehydration, isomerization, and polymerization steps that are still poorly understood. Similar and additional amounts of the same flavor components found in the cured leaves are formed during smoking.

## 4. Biological data related to transferred unchanged sugars

### 4.1. Transfer of unchanged sugars to mainstream smoke

Carbohydrates have very low vapor pressures and are characterized by thermal and oxidative lability at the temperatures occurring in a burning cigarette (Baker et al., 2005). Indeed, very small yields (about 0.5% [range 0.4–0.8%]) of uniformly ^14^C-labeled glucose and sucrose were found to transfer unchanged from Burley tobacco into mainstream smoke (Gager et al., 1971a), supporting the results of an earlier study (Kobashi and Sakaguchi, 1959). Based on similarity in the structures of glucose, fructose and sucrose, it is expected by analogy that the unchanged transfer of fructose to smoke will be low as well.

If approximately 0.5% of the sugar added to cigarette tobacco is transferred unchanged to smoke, the daily dose of sugar from smoking would be approximately 0.008 g per person per day under the worst case assumptions given in Table 1. This is negligible, e.g. in terms of caloric uptake, when compared with a daily dietary sugar intake in Western countries in the order of 100 g per person per day (Gibney et al., 1995).

**Table 1:** Estimate of ingredient sugar uptake (worst case assumptions) by smoking American-blend cigarettes.

Parameter	Estimate
Number of cigarettes per day§	40 cig.
Maximum use level for all sugar sources	5%
Tobacco weight	0.8 g/cig.
Transfer of unchanged sugars	0.5%
Bioavailability (deposition)	100%
Dose per cigarette	0.2 mg
Daily dose	8 mg

For risk extrapolations, worst case assumptions were made: sucrose, glucose, or fructose, respectively, used as if representing total sugar doses; local deposition on tongue or in lungs as if total dose would be deposed at either site.

§ 95 percentile (Waingrowet al., 1968).

### 4.2. Absorption, distribution, and metabolism of sucrose, fructose, and glucose after inhalation

During smoking, minor unchanged portions of sucrose, glucose, and fructose are inhaled and exposed to the respiratory tract. Sucrose cannot be taken up or hydrolyzed in pulmonary epithelial cells (Ricard et al., 2000). If not cleared by mucociliary clearance, it passively leaks through the alveolar wall, distributes systemically, and is excreted unchanged in the urine (Martindale, 1993). Glucose and fructose are taken up by respiratory tract tissues and used for intermediary metabolism. In tracheal and pulmonary experimental systems, active glucose uptake was demonstrated to be regulated by nutritional and hormonal mechanisms (Das et al., 1985). Glucose is actively removed from the airway lumen against a 10-fold higher glucose plasma concentration gradient (Baker et al., 2006a). Since no such physiological regulation of fructose utilization in lungs has been observed, the uptake of fructose into alveolar epithelial cells is assumed to be by gradient-driven facilitated diffusion, similar to fructose uptake in intestinal epithelial cells.

### 4.3. Physiological properties of sugars

Apart from the nutritional value, sugars exert the sensorial cue of a sweet taste on the tongue. The thresholds at which sweet tastes are recognized for sucrose, fructose, and glucose are relatively high, i.e. in the order of 10 g/l (Soldo et al., 2003; Pasquet et al., 2006), and these thresholds do not seem to be influenced to a relevant degree by smoking (Mullings et al., 2010). If the entire amount of unchanged sugars taken up by smoking (Table 1) were to dissolve in the saliva (daily production 1–1.5 1, Thews et al., 1989), this would result in a maximum average concentration of 0.01 g/l, which is three orders of magnitude below the threshold at which a sweet taste is detected. The volume of saliva covering the sensory area of the tongue at a given time would need to be as low as 20 μl in order to reach a level at which a sweet taste could be detected with the 0.2 mg of sugar absorbed from smoking one cigarette with added sugar, assuming full dissolution in the saliva and no transfer to lower parts of the respiratory tract. Thus, it is unlikely that the sugar transferred unchanged to smoke would elicit a sweet taste sensation in the smoker. Since sweet taste sensation and palatability are necessary requirements of any central reward mechanism for sugars (Barbano and Cador, 2007; Olszewski and Levine, 2007), there is no practical possibility of the small amounts of unchanged sugars in smoke to contribute to the initiating and addicting potency of smoking, as was previously suggested (Talhout et al., 2006; Rabinoff et al., 2007).

The above rationale renders a sweet sensation by unchanged sugars used as cigarette tobacco ingredients very unlikely. Nevertheless, the use of sugars positively adds to the overall sensory perception of tobacco smoke (Leffingwell, 1976; Fisher, 1999; Rodgman, 2002; Talhout et al., 2006). In the subjective evaluation of cigarette smoke taste, trained panels have used the term “sweet” as a descriptor (Baker, 2007). However, this is meant as a pleasant, mild, non-harsh quality, quite different from the taste quality of a sweet receptor sensation. It is unclear to what extent sugar-dependent changes in mainstream smoke composition might elicit responses at taste receptors independent of the role of the parent ingredients.

### 4.4. Toxicological properties of sugars

Sucrose and invert sugar are GRAS and can be used in food without limitation (US Food and Drug Administration, 2000a,b). The three sugar compounds exert *in vitro*, acute, and chronic toxicities only at exaggerated exposures (American Congress of Governmental Industrial Hygienists, 1991). A number of chronic diseases has been associated with sugar-related malnutrition (e.g. Basciano et al., 2005; Michaud et al., 2005). Sugar-rich food, total sucrose intake, ratio of sucrose to dietary fiber, and glycemic index were associated with an increased risk for lung cancer in a case-control study (De Stefani et al., 1998), while in another study, no such relationship was seen (Burley, 1998). Potential underlying mechanisms for the chronic diseases related to malnutrition- or disease-related sugar imbalances include glucotoxicity, which depends on the shift of glucose metabolism from the glycolytic pathway to minor forms of glucose metabolism, including increased oxidative stress (Federici and Lauro, 2005), and the formation of advanced glycation end products from reducing sugars, such as fructose (Gaby, 2005).

All of these effects, even those related to the respiratory tract, are not relevant to the minute sugar uptake from smoking. Nevertheless, the uptake of sugars via inhalation needs to be specifically assessed, as elevated glucose concentrations in the airway epithelial lining fluid have been associated with accelerated growth of pulmonary pathogens and pulmonary inflammation (Baker et al., 2006a). Occupational exposure to sucrose is only regulated based on its nuisance dust character at 5 mg/m^3^ (US Occupational Safety and Health Administration, 2010), which would lead to a daily sucrose uptake of approximately 50 mg of sucrose, which is above the daily dose of sugars reaching the smoker's respiratory tract if estimated under worst case conditions (Table 1). Glucose levels in airway secretions are approximately one tenth of the blood glucose levels (Baker et al., 2006a), i.e. at about 100mg/l. With an estimated volume of lung epithelial lining fluid of 30 ml (Rennard et al., 1986), 0.2 mg of glucose stemming from smoking one cigarette would result in a local transient increase in concentration of 7mg/l, i.e. an increase of 7% above baseline. This increase is within the physiological variation expected for epithelial lining fluid glucose levels due to diet-induced changes in blood and, therefore, would not be expected to increase the risk of local pulmonary adverse effects associated with hyperglycemia. Fructose levels in the epithelial lining fluid are similar to those in the blood, i.e. 10mg/l (Gaby, 2005), since there is no active fructose transport in the lungs. Addition of 7 mg/l from smoking one cigarette, calculated under worst case assumptions, is in the same order of magnitude as the physiological fructose level in the epithelial lining fluid. Blood fructose levels can increase by fivefold or more after a higher dietary dose of fructose. Therefore, the estimated increase of pulmonary fructose from smoking is within the range of what can be expected by transiently increasing the blood fructose levels by dietary uptake.

Sugars have also been approved for use as excipients for pulmonary drug delivery, such as glucose or lactose in dry powder inhalers (Kohlhäufl et al., 2004; Pilcer and Amighi, 2010). Lactose, a disaccharide very similar in structure to sucrose, is well recognized as a safe pharmaceutical excipient for use in oral or inhalation formulations and is also not likely to constitute any significant toxicological hazard to man (Baldrick and Bamford, 1997). The use of sugars as inhalation excipient supports the notion that the unchanged sugar that may be inhaled with smoking does not contribute any toxicity.

## 5. The effect of sugars on mainstream smoke composition

The most straight-forward approach to address the effects of sugars used as ingredients on mainstream smoke composition is to investigate the overall composition of mainstream smoke generated from research cigarettes with or without sugars applied to tobacco. Direct precursor-product relationships, which may be potentially relevant for smoking, can be qualitatively addressed by pyrolyzing the ingredient as a neat compound or in combination with other tobacco constituents or tobacco itself. One way of studying quantitative precursor-product relationships in the tobacco matrix is the use of radiolabeled precursors. Further information can be obtained by comparing market cigarettes that may or may not include the ingredient under investigation *(vide infra)*.

### 5.1. Pyrolysis experiments with sugars

Direct ingredient-product relationships have been investigated in more or less controlled pyrolysis experiments of variable design, mostly in the absence of tobacco. Pyrolysis is the breakdown of larger compounds to smaller ones caused by exposure to heat, sometimes in the presence of reactive gases such as oxygen. Pyrolytic products of tobacco constituents or ingredients can also form compounds that have molecular weights larger than the precursor by a process known as pyrosynthesis. In addition, when oxygen is present, oxidative reactions can occur such as combustion. During smoking, most tobacco is combusted to carbon dioxide and water or incompletely combusted to carbon monoxide or less oxidized smoke constituents (Green and Rodgman, 1996).

**Figure 2:**
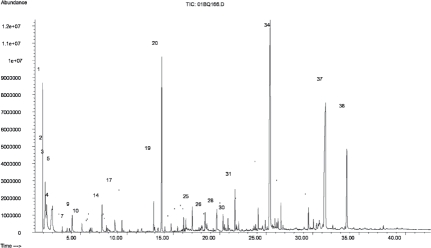
Total GC-MS ion current finger-print chromatogram obtained from the pyrolysis of sucrose in air. For compound identifications, see Table 2. The pyrolysis unit was programmed to 1000°C with a three-step temperature program (400, 700, and 1000°C) with a 10-s hold time at each temperature. Comparison to data published elsewhere (Baker et al., 2005) suggest that yields of pyrolysis products are in the order of 1 μg per mg sugar pyrolyzed in this type of experiment.

Glucose, fructose, sucrose (Table 2, Figure 2), invert sugar, and also cellulose produce essentially the same compounds upon pyrolysis, and any differences observed in the pyrolysates of the carbohydrates are quantitative rather than qualitative (Sanders et al., 2002; Baker et al., 2005). This is an expected result given the close chemical and structural similarity of these carbohydrates. Different patterns of carbonyl compounds were found depending on the sugar type investigated (Baker et al., 2005; Talhout et al., 2006). The pyrolysis products from the mono- and disaccharides are also products of the pyrolysis of tobacco itself, since tobacco contains approximately 10% of the polysaccharide cellulose (Leffingwell, 1999) and, except for uncased cured Burley tobacco, high concentrations of sugars as well (Sanders et al., 2002; Baker et al., 2005). Major pyrolysis products from glucose, fructose, and sucrose are furans, including furfural and 5-hydroxymethylfurfural, as well as acetic acid and levoglucosan. Furfural yields were higher than those of formaldehyde in pyrolysis experiments. Formaldehyde predominately stems from the hydroxymethyl moiety of sugars. Pyrolysis of cellulose yields a greater percentage of low molecular weight ketones and aldehydes, such as acetaldehyde and hydroxyacetaldehyde, relative to glucose, fructose and sucrose (Sanders et al., 2002). Pyrolysis yields of acrolein from various sugar types are inconsistent, although acrolein does appear to be a pyrolysis product from sucrose as well as from monosaccharides (Baker et al., 2005). The presence of other materials in such pyrolysis experiments, such as tobacco, ammonia compounds, or metal salts was shown to change the quantitative composition of the respective pyrolysates (Torikai et al., 2005). Carbonyl compounds stemming from the pyrolytic degradation of sugars may react with ammonia compounds (Baker et al., 2006b), and the pyrosynthetic formation of aromatic hydrocarbons was also described under certain conditions (Britt et al., 2004).

**Table 2:** Compounds qualitatively identified in a sucrose pyrolysis experiment according to Figure 2.

Peak number	Compound
1	Air
2	Carbon dioxide
3	Formaldehyde
4	Water
5	Acetaldehyde
6	Furan
7a	Mixture of acrolein and
7b	2-propanol
8	Acetone
9	Pyruvaldehyde
10	2-methylfuran
11	2-butanone
12	3-buten-2-one
13	Diacetyl
14	Glycolaldehyde
15	2,5-dimethylfuran
16	Formic acid
17	Acetic acid
18	1-hydroxy-2-propanone
19	3-furanone
20	Furfural
21	2-furanmethanol
22	2-acetylfuran
23	2(3H)-dihydro-4-hydroxy-2-3(H)furanone
24	2-hydroxycyclopent-2-en-1-one
25	5-methyl-2-furfural
26	1,3-dihydroxy-2-propanone
27a	Mixture of 2-hydroxy-3-methyl-2-cyclopenten-1-one and
27b	furanone
28a	Mixture of 2,5-dimethyl-4-hydroxy-3-(2H)furanone and
28b	2,4-hexanedione
29	Methylfuroate
30	5-methyl-1,3-benzenediol
31	2,3-dihydro-3,5-dihydroxy-4H-pyran-4-one
32	3,5-dihydroxy-2-methyl-4H-pyran-4-one
33	1,4:3,6-dianhydro-α-D-glucopyranose
34	5-(hydroxymethyl)-2-furfural
35	1,5-anhydro-4-deoxy-D-glycero-hex-1-en-3-ulose
36	An anhydro-sugar
37	Levoglucosan
38	1,6-anhydro-β-D-glucofuranose

The extrapolation of the results of pyrolysis model experiments to mainstream smoke chemistry is difficult because no model can completely replicate the complex chemical and physical processes that occur under real-life smoking conditions or even those that occur during machine-smoking. During smoking, the chemical transformations that carbohydrates undergo are influenced by many factors, including time-dependent local temperature changes, the concentration profile of oxygen and other participating substances at and behind the burning zone, and pyrosynthetic reactions involving two or more tobacco or smoke constituents. Thus, although pyrolysis experiments may provide qualitative information on potential reactions, more relevant information regarding the fate of sugar ingredients in tobacco products and their reaction products during smoking is obtained in cigarette-smoking experiments, i.e. in the right matrix and with standardized smoking and quantitative analytical protocols. Other authors concluded that the pyrolysis of the neat ingredient by itself is not sufficient for assessment (Hahn and Schaub, 2010).

### 5.2. Pooled quantitative chemical analyses of research cigarettes with and without sugars applied as tobacco ingredients

Over the past several years, three publications appeared covering a series of chemical-analytical studies on the mainstream smoke composition of research cigarettes with and without sugars applied as single tobacco ingredients that reported the yields of a broad series of toxicologically relevant constituents (Table 3) (Baker, 2006; Roemer et al., 2010; Coggins et al., 2011). Various forms of sugars were investigated, including sucrose and invert sugar, but also white and brown sugar, glucose, fructose, high fructose corn syrup (HFCS), honey, and molasses. All of these studies used tobacco blends representative of American-blend cigarettes to produce research cigarettes that were cased with various levels of the respective sugar ingredient. In most studies, the highest application levels exceeded the maximum application levels of sugars in commercial cigarettes (for overview, see Talhout et al., 2006) in order to improve the ability to detect differences in the smoke composition or the toxicological activity of the research cigarettes with and without sugar application. The cigarettes were machine-smoked under ISO standard conditions (International Organization for Standardization, 2000), and, in one study, under Health Canada intense (HCI) conditions (Health Canada, 2000) in parallel. The TPM yields for the research cigarettes included in the current analysis varied between 6 and 18 mg per cigarette using the ISO smoking regimen, and should thus address much of the variability seen for marketed cigarettes. ISO nicotine yields varied from 0.5 to 1.2 mg per cigarette. A set of more than 35 generally accepted and toxicologically relevant smoke constituents was analyzed in these studies, which has been developed in similar compositions on the basis of the so-called Hoffmann analytes (US Consumer Product Safety Commission, 1993; Health Canada, 2000).

**Table 3:** Regression analyses of individual and pooled chemical-analytical data on the relationship of mainstream smoke constituent yields (nicotine-based) and sugar addition levels in research cigarettes.

	Statistical significance of slopes and change at 5% application														
	
	ISO data												HCI data		
		
	Individual studies *&*								Pooled studies				Individual		Pooled
				
Constituent	1	2	3	4	5	6	7	8	Slope	*p* value	R2	Change§	7-HCI	8-HCI	Slope
Tar	=	=	=	=	=	=	=	=	=	0.9491	-	-	=	=	=
Carbon monoxide	=	=	=	=	=		=	=	=	0.1804	-	-	=	=	=
Formaldehyde	=	↑	=	↑	↑	↑	=	=	↑	0.0005	0.24	25%	=	=	=
Acetaldehyde	=	↑	↑	=	=	=	=	↑	=	0.3826	-	-	=	=	=
Acrolein	=	↑	=	↑	=	↑	=	=	↑	0.0009	0.22	10%	↑	=	=
Propionaldehyde	=	=	↑	↑	=	↑	=	↑	=	0.6376	-	-	↑	=	↑
n-Butyraldehyde	-	-	-	-	=	=	=	↑	=	0.5435	-	-	=	=	=
Crotonaldehyde	-	-	-	-	=	=	=	↑	=	0.1022	-	-	=	=	=
Acetone	-	-	-	-	↑	=	=	↑	=	0.1234	-	-	=	=	=
2-Butanone	-	-	-	-	↑	=	=	↑	↑	0.0045	0.29	9%	=	=	=
1,3-Butadiene	=	=	=	↑	-	-	=	↑	=	0.6112	-	-	=	=	=
Isoprene	=	=	=	=	-	-	=	↑	↑	0.0065	0.25	12%	=	=	=
Acrylonitrile	↑	=	=	↑	-	-	=	=	=	0.2444	-	-	=	=	=
Hydrogen cyanide	=	=	↑	↑	-	-	=	=	=	0.6849	-	-	=	=	=
2-Nitropropane	=	=	=	=	-	-	-	-	=	0.6466	-	-	-	-	-
4-Aminobiphenyl	=	=	=	=	-	-	=	=	↓	0.0124	0.22	−21%	=	=	=
o-Toluidine	↓	↓	=	=	-	-	-	-	=	0.5124	-	-	-	-	-
2-Naphthylamine	=	=	=	=	-	-	-	-	=	0.8311	-	-	-	-	-
o-Anisidine	-	-	=	=	-	-	-	-	=	0.2250	-	-	-	-	-
Nitrogen oxides	=	=	=	=	-	-	↓	=	=	0.5313	-	-	=	=	=
Benzene	=	↑	↑	↑	-	-	=	↑	↑	0.0011	0.34	9%	=	=	=
Toluene	↑	↑	↑	-	-	-	=	↑	↑	<0.0001	0.51	9%	=	=	=
Styrene	-	-	-	↑	-	-	=	=	=	0.0039*	-	-	=	=	↑
N-Nitrosodimethylamine	-	-	=	↑	-	-	-	-	↓	0.0003	0.74	−12%	=	=	=
N-Nitrosopyrrolidine	=	=	=	↓	-	-	-	-	-	0.0902	-	-	-	-	-
N-Nitrosonornicotine	=	=	=	↓	-	-	↓	=	↓	<0.0001*	0.69	−12%	=	=	=
NNK	=	=	=	=	-	=	=	=	0.4278*	-	-	-	-	-	-
Phenol	=	=		↓	-	-	=	=	=	0.4072	-	-	=	=	=
Catechol	=	=	=	=	-	-	=	=	=	0.1749	-	-	=	=	=
Benzo[*a*]anthracene	=	=	=	=	-	-	-	-	=	0.9136	-	-	-	-	-
Benzo[*b*]fluoranthene	=	=	=	↑	-	-	-	-	=	0.6700	-	-	-	-	-
Benzo[*j*]fluoranthene	-	-	=	↑	-	-	-	-	=	0.8542	-	-	-	-	-
Benzol[*a*]fluoranthene	-	-	=	↑	-	-	-	-	↑	0.0044	0.57	7%	-	-	-
Benzo[*a*]pyrene	=	=	=	↑	-	-	=	=	=	0.2168	-	-	=	=	=
Indeno[l,2,3-*cd*] pyrene	↑	=	=	↑	-	-	-	-	=	0.2400	-	-	-	-	-

Sugars used as ingredients in individual studies:

1: Sucrose (Coggins et al., 2011).

2: Invert sugar (Coggins et al., 2011).

3: Honey (Coggins et al., 2011).

4: High fructose corn syrup (Coggins et al., 2011).

5: White and brown sugar, invert sugar (Baker, 2006; test cigarettes D2 to D5, D8, and D10 vs. control cigarette D1).

6: Brown sugar, invert sugar, honey, glucose, fructose (Baker, 2006; test cigarettes E2 to E9, E11, and E13 vs. control cigarette E1).

7: Sucrose, research cigarettes yielding 6 mg ISO tar/cig., machine-smoked under ISO and HCI conditions (Roemer et al., 2010).

8: Sucrose, as in 7 but with research cigarettes yielding 10 mg ISO tar/cig. (Roemer et al., 2010).

↑, ↓: Slopes of regression analysis (GraphPad software) statistically significantly different from zero (increase, decrease); = : slopes of regression analysis not statistically significantly different from zero; -: not done/not applicable; R^2^: regression coefficient.

§ Change at a usual maximum use level of 5% sugar addition relative to control without sugar use.

* Two different pools of data from different laboratories, lowest *p* value given.

ISO smoking conditions: puff volume, duration, and frequency of 35 ml, 2 s, and lmirr^1^, respectively (International Organization for Standardization, 2000).

HCI (Health Canada intense) smoking conditions: puff volume, duration, and frequency of 55 ml, 2 s, and 2min∼^1^, respectively, and blocked filter ventilation holes (Health Canada, 2000).

In order to obtain added statistical power by a larger sample size, the results of all relevant studies obtained under ISO smoking conditions were pooled for the individual constituents, regardless of the sugar type examined in the particular studies. This was possible because of the similarity in cigarette construction, smoking conditions, and analyses. Molasses was excluded from this pool because it was considered to contain significant concentrations of materials other than sugars. The pooled data were analyzed by linear regression as a function of the sugar application level, and the resulting slopes were statistically tested for difference from a zero slope (Table 3). This approach should reduce the number of false positive statistical results from the several hundred individual statistical tests underlying this pool of data and should be more discriminative than the analysis of individual studies, if the inter-study variabilities are small. The approach of pooling the results obtained using different types of sugars was corroborated by the lack of differences seen in a direct comparison of the smoke composition generated from research cigarettes with corn syrup/invert sugar or, alternatively, high fructose corn syrup (Stavanja et al., 2006).

On a nicotine basis, there was no effect by the sugar application on the yields of the leading quantitative smoke parameters for the particulate and gas/vapor phases, i.e. tar and carbon monoxide, for a range of application levels up to approximately 10% (Table 3). Under ISO smoking conditions, the nicotine-based yields of most smoke constituents in the pooled analysis, such as those of acetaldehyde, 1,3-butadiene, phenol, 4-(methylnitrosamino)-1-(3-pyridyl)-1-butanone (NNK), or benzo[*a*]pyrene, did not change in a statistically significant manner with increasing sugar application levels (Table 3). However, the nicotine-based yields of seven constituents increased in a statistically significant manner, i.e. formaldehyde, acrolein, 2-butanone, isoprene, benzene, toluene, and benzo[*k*]fluoranthene, while those of N-nitrosodimethylamine, N-nitrosonornicotine, and 4-aminobiphenyl decreased. These results are in broad agreement with the model pyrolysis results, i.e. an increased yield in some carbonyl constituents and vice versa a decrease in some nitrogen-containing constituents, the precursors of which might have been trapped by the excess carbonyl compounds. No nitrogen-containing constituent increased in yield, which is also attributable to the replacement of nitrogen-containing tobacco material with the carbohydrate ingredient. There is no ready explanation why one of the three benzo-fluoranthene isomers would increase but not the others. For benzo[*a*] pyrene, used as a surrogate for the group ofpolycyclic aromatic hydrocarbons in many analyses, there was also no statistically significant change.

**Figure 3:**
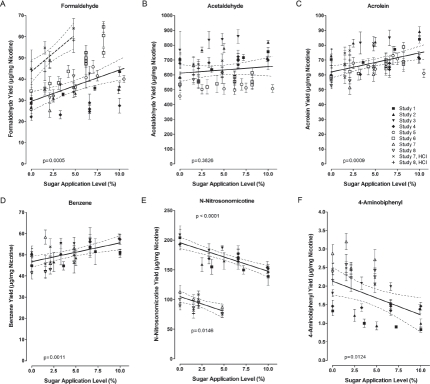
Yields (nicotine-based) of formaldehyde (A), acetaldehyde (B), acrolein (C), benzene (D), N-nitrosonornicotine (E), and 4-aminobiphenyl (F) in the mainstream smoke of research cigarettes of pooled studies with research cigarettes with varying sugar application levels. Linear regression (solid lines) with 95% confidence limits was performed on yield data obtained with ISO machine-smoking. For formaldehyde, an additional linear regression (dashed line) was derived for yield data obtained under HCI smoking conditions. The HCI data for the other constituents fit well to the set of ISO data. M ± SD for individual data points (SDs by non-weighted error propagation). The legend in graph C applies to all graphs in Figure 3; for study references, see Table 3.

Under the more intense HCI machine-smoking conditions, most of the above-discussed differences were no longer apparent (Table 3). After pooling the results obtained with the two research cigarettes types, slopes with statistically significant increases were only seen for the nicotine-based yields of formaldehyde, propionalde-hyde, and styrene (Table 3). Most of the nicotine-based yields obtained under HCI conditions fitted well with those obtained under ISO conditions (Figure 3), confirming the validity of the current results and the applicability of the current interpretations to the broad spectrum of human smoking conditions. Only the nicotine-based formaldehyde yields seemed to be approximately 50% higher under HCI than under ISO conditions, but with a similar percentage of increase at 5% compared to 0% sugar application (Figure 3A). Thus, the sugar application-dependent effects observable under ISO machine-smoking conditions tend to be less pronounced under more intense smoking conditions, which is probably due to more complete combustion during smoking. This is in line with the general observation of less difference among test results for various non-clinical tests of smoke for samples generated under intense versus ISO conditions (Roemer and Carchman, 2011).

#### 5.2.1. Formaldehyde

The most pronounced change with increasing sugar application levels in the pooled data were for formaldehyde (Figure 3A). At a use level of 5%, this correlation reveals a maximum increase by 25% over the control with no added sugar. About a quarter of the observed variation in formaldehyde formation can be ascribed to the sugar application *(R^2^*, Table 3). Further variation may be associated with potential differences in the series of research cigarettes used in the individual studies and with analytical variations both within and between the laboratories. Formaldehyde is also formed from tobacco matrix components, such as cellulose, and is higher in the first puff of mainstream smoke than in subsequent puffs (Parrish and Harward, 2000; Baker, 2006). Correlations of formaldehyde with other smoke constituents may also be influenced by its reactivity with components of the tobacco rod and in the smoke (or in the sampling device). The overall formaldehyde yield in commercial cigarettes is correlated with the respective tar yield at less intense but not at HCI machine-smoking conditions (data from Gregg et al., 2004; Counts et al., 2005; Hyodo et al., 2007).

The relationship of tobacco types and sugar and ammonia content to formaldehyde yields was investigated in a puff-wise analysis of single-blended cigarettes, i.e. research cigarettes made from only one type of tobacco. Burley, Virginia, and Oriental tobaccos yielded 1.0, 3.9, and 7.7 μg of formaldehyde per puff, respectively, with corresponding reducing sugar contents in the tobaccos of 2.2, 8.3, and 15.9% (Parrish and Harward, 2000). If the Virginia tobacco was expanded by carbon dioxide in the presence of ammonia, the formaldehyde yield dropped to 2.0 μg/puff, indicating that its yield depended both on the availability of sugars providing the carbonyl functions and of nitrogen-containing components in tobacco or smoke scavenging these carbonyls. In an analytical study on the mainstream smoke of research cigarettes with various combinations of tobacco ingredients, formaldehyde was found to increase by 77% (nicotine-based) in the group containing corn syrup (at 6.3%) among other ingredients, but it only slightly increased in the group containing invert sugar and sucrose (at a total of 13%) in another combination (Rustemeier et al., 2002), again indicative of interactions between cigarette or smoke components leading to non-proportional sugar-formaldehyde relationships. Significantly increased yields of formaldehyde (up to 88%, nicotine-based) were also observed with another series of ingredient mixtures with sugar application levels of up to 10.5% (Baker et al., 2004b).

#### 5.2.2. Acetaldehyde

In the current pooled analysis, there was no statistically significant effect on acetaldehyde yields with increasing sugar application levels (Figure 3B), although such increase was determined in three of the eight individual studies (Table 3). There was also no sugar-related effect if research cigarettes were smoked under HCI conditions.

This overall lack of effect is consistent with the results of a previous review which concluded that the sugars used as ingredients would not lead to an increase in the yield of acetaldehyde in commercial cigarettes (Seeman et al., 2002). This conclusion was questioned in a more recent review (Talhout et al., 2006) on the basis of two particular studies, in which sugars were added to experimental cigarettes at levels of up to 20%, resulting in increases in aldehyde yields per cigarette (Zilkey et al., 1982; Shelar et al., 1992). However, these cigarettes were so heavily loaded with sugars that the burning characteristics changed leading to an increase in tar yield as well. If based on tar yield, the relative yield of acetaldehyde did not change in one study (Zilkey et al., 1982; without using those cigarettes containing a tobacco substitute or with charcoal filtration) or only changed in one of two sets of experimental cigarettes (Shelar et al., 1992) as a function of the sugar application level. Furthermore, it remains questionable if results obtained at 16–20% sugar application levels can indeed be meaningfully back-extrapolated and would thus be relevant for the maximum 5% levels used in most commercial cigarettes. The lack of a relevant acetaldehyde effect in the above two studies with normalization to tar yields is in line with the results of benchmark studies evaluating the smoke composition of major brands of cigarettes in a given market, showing that the acetaldehyde yield was found to be tightly correlated with the tar yield (data from Gregg et al., 2004; Counts et al., 2005; Hyodo et al., 2007), while no correlation with sugar content was found for the acetaldehyde yield, neither absolute per cigarette nor relative to tar yield (Phillpotts et al., 1975; Seeman et al., 2003). In a study using ingredient combinations including sugars, acetaldehyde yields relative to tar slightly decreased in the groups using sugars as ingredients, while the same data relative to nicotine were found to slightly increase (by up to 11%, not dependent on sugar application level) (Rustemeier et al., 2002). A trend to slightly higher acetaldehyde yields (by up to 23%, nicotine-based, not dependent on sugar application level) was also observed in another ingredient mixture study with sugar application levels up to 10.5% (Baker et al., 2004b). Thus, although few individual studies may have found sugar-related increases in acetaldehyde yield, this is not apparent in most others, in particular in those using cigarettes with designs that are closer to commercial cigarettes. In addition, acetaldehyde yields need to be normalized to those of tar or nicotine to correct for potential changes in burning characteristics in case of high experimental sugar applications.

In an experimental study using radiolabeled glucose and sucrose added to research cigarettes, up to 0.06% of the label was found in acetaldehyde (Gager et al., 1971b), which at a sugar application level of 5% (40 mg per cigarette) would translate to an ingredient-related contribution to the overall acetaldehyde yield of approximately 5%, assuming an average overall yield of acetaldehyde of approximately 500 μg per cigarette.

Taken together, sugars used as ingredients do not produce greater yields of acetaldehyde in mainstream smoke than are produced from tobacco itself, which they replace as an ingredient on a weight-for-weight basis (Seeman et al., 2002). This conclusion is important considering the suggestions that sugars could be added with the intention to increase the yield of acetaldehyde so as to increase the addictive potency of cigarette smoke by interacting with nicotine or by the formation of derivatives with biogenic amines (Belluzzi et al., 2005; Talhout et al., 2006; Rabinoff et al., 2007; Talhout et al., 2007; European Commission Health and Consumer Protection Directorate-General Scientific Committee on Emerging and Newly Identified Health Risks, 2010).

#### 5.2.3. Acrolein and 2-Butanone

The nicotine-based yield of acrolein increased slightly with increasing sugar application levels, resulting in an additional yield of 10% at a 5% sugar application level compared to the control with no added sugar (Table 3, Figure 3C). Statistically significant increases were found in three of the eight individual studies under ISO conditions and also in one case under HCI conditions. As was argued in the report of one of these studies, there is an appreciable long-term analytical variability of similar magnitude as the sugar-related effect (Baker, 2006). If sugars were used in a mixture of ingredients, there was a slight increase in nicotine-based acrolein yields (up to 18%, not dependent on sugar application level) but a decrease if related to tar yield (Rustemeier et al., 2002). A trend to increased acrolein yields (up to 24%, nicotine-based, independent of the sugar application level) was also observed in another ingredient mixture study with sugar application levels of up to 10.5% (Baker et al., 2004b).

The nicotine-based yield of another C4-carbonyl constituent, i.e. 2-butanone or methyl ethyl ketone, increased slightly but statistically significantly with increasing sugar application levels, resulting in an additional yield of 9% at a 5% sugar application level compared to the control with no added sugar when smoked under ISO conditions (Table 3). This effect was not seen under HCI conditions. A numerical increase in 2-butanone yields of the same magnitude (nicotine-based, independent of the sugar application level) was also seen in the smoke generated from research cigarettes with ingredient mixtures containing sugars at application levels of up to 10.5% (Baker et al., 2004b).

In a study using uniformly ^14^C-labeled glucose and sucrose as ingredients, less than 0.01% of the label was recovered as acrolein, and 0.02–0.03% of the radiolabel was found as 2-butanone (Gager et al., 1971b). The most important parameter for the yield of both constituents is the overall smoke yield of a cigarette, as demonstrated by the tight correlation of acrolein yields with those of tar or carbon monoxide in various series of commercial cigarettes (data from Gregg et al., 2004; Counts et al., 2005; Hyodo et al., 2007).

#### 5.2.4. Volatile Hydrocarbons

There was a rather consistent increase in the nicotine-based yields of benzene (Figure 3D) and toluene with increasing sugar application levels among the ISO smoking studies entering the pooled analysis, although the overall increases in yield at the 5% sugar application levels were relatively low (9%, Table 3). If tested under HCI conditions, there was no sugar-related effect observed for either of these two constituents but there was for styrene, a constituent with similar chemical structure.

Increases in nicotine-based yields of benzene and toluene of up to 13 and 25%, respectively, were also observed in one study on research cigarettes with added tobacco ingredient mixtures containing sugars (Rustemeier et al., 2002), but not in another (Baker et al., 2004b). The observations in the first ingredient mixture study may simply be due to the replacement of nicotine-containing tobacco with the ingredient mixture, thus lowering the normalization basis for the specific constituent yields, because these effects were absent if yields were related to tar. Such effects may of course also be related to other components of the ingredient mixtures than sugars. Again, the yield of these two smoke constituents correlated well with tar or carbon monoxide yields in the smoke of commercial cigarettes (data from Gregg et al., 2004; Counts et al., 2005; Hyodo et al., 2007). Although it may be conceivable that benzene could be formed from the C6 structure of mono-saccharides, less than 0.01% of radiolabeled glucose or sucrose was found to be converted to benzene when added to experimental cigarettes (Gager et al., 1971b).

A 12% increase of the nicotine-based yield of isoprene was observed in the pooled analysis of the data generated under ISO conditions. This was not observed under HCI conditions.

#### 5.2.5. N-Nitrosamines

The increased yield seen for some carbonyl constituents may be related to the decreased yields for some nitrogen-containing mainstream smoke constituents, because some carbonyl compounds may trap nitrogen-containing precursors of these constituents. The nicotine-based yields of N-nitrosodimethylamine and N-nitrosonornicotine were found to decrease with increasing sugar application levels, and approximately 70% of the variability seen for the two constituents can be explained by the sugar application (Table 3). At the 5% application level, this would result in decreased yields of 12% compared to a control without added sugars. No effect was found for N-nitrosonornicotine, if smoke was generated under HCI conditions. Interestingly, there is no trend for the NNK yields (nicotine-based), which might be explainable by the higher proportion of NNK preformed in the tobacco versus its formation during smoking in comparison to N-nitrosonornicotine (Moldoveanu and Borger Ding, 2008). There were clearly two distinct classes of yields for the tobacco-specific N-nitrosamines (Figure 3E). This may be related to diverging analytical methodologies but may also reflect different generations of research cigarettes with lowered preformed contents of tobacco-specific N-nitrosamines in the tobacco of more recent productions (Boyette and Hamm, 2001).

Decreases for the nicotine-based yields of both N-nitrosonornicotine and NNK were found in the smoke of the research cigarettes with sugar-containing mixtures (Rustemeier et al., 2002). In another ingredient mixture study, N-nitrosonornicotine yields were more decreased than NNK yields (Baker et al., 2004b). For commercial cigarettes, there is a categorical difference between American-blend and Virginia-type cigarettes due to the higher nicotine content in Burley tobacco, and the yields of the tobacco-specific N-nitrosamines in general correlate less with those of other constituents (data from Gregg et al., 2004; Counts et al., 2005; Hyodo et al., 2007).

#### 5.2.6. 4-Aminobiphenyl

The nicotine-based yields of several aromatic amines decreased with increasing sugar application levels, an effect which was statistically significant for 4-aminobiphenyl (Table 3, Figure 3F). At a 5% sugar application level, this would result in a 21% decrease of the 4-aminobiphenyl yield. This is in line with the above-mentioned scavenging of nitrogen-containing intermediates by carbonyls, which are formed in slightly increased amounts during the pyrolysis of the sugars. The effect was not seen if tested under HCI smoking conditions. A trend to decreased yields of 4-aminobiphenyl (up to 19%, nicotine-based) was also observed for the smoke from research cigarettes with ingredient mixtures containing sugars (Baker et al., 2004b). The absolute yields of 4-aminobiphenyl and the other aromatic amines are relatively low (low ng range per mg nicotine) compared to those of the above-mentioned constituents, such as the aldehydes (high μg range per mg nicotine). As for other nitrogen-containing smoke constituents, there are categorical differences between American-blend and Virginia-type cigarettes due to the higher nicotine content in Burley tobacco (data from Gregg et al., 2004; Counts et al., 2005; Hyodo et al., 2007).

### 5.3. Additional constituents of mainstream smoke

Additional studies were performed to characterize the effect of using sugars as tobacco ingredients, which analyzed less comprehensive lists of smoke constituents compared to the above experiments. Pyrolysis experiments had suggested the formation of furfural from sugars. An almost threefold increase in furfural yield (nicotine-based) was indeed found in chemical-analytical studies on the smoke from Burley cigarettes with fructose and/or glucose at application levels up to 18% (Thornton and Massey, 1975). Since Burley is practically devoid of sugars, this study also showed that other tobacco components also give rise to the formation of furfural. Cigarettes made of Virginia tobacco with high natural sugar content (17%) also had about threefold higher furfural yields than the Burley cigarettes, which further increased by glucose application. This study also confirmed the general increase in carbonyl constituents by the presence of sugars in the tobacco blend.

With radioactive labeling, further products of glucose and sucrose used as cigarette ingredients were identified in mainstream smoke (Gager et al., 1971a,b). These include furan, alkylated derivatives of furan, and acetonitrile. None of these constituents was found at recoveries of more than 0.07% of the added tracer. Some constituents found in this tracer study were also included in the above pooled analysis of mainstream smoke constituents, but did not show a statistically significant increase with increasing sugar application levels in the pooled analysis. These constituents include acetone and crotonaldehyde. Acetone had the highest radiochemical yield of 0.1% of the added tracer identified, which at a sugar application level of 5% (40 mg per cigarette) would translate to a sugar-related contribution to the overall acetone yield of approximately 10–15%, assuming an average overall yield of acetone of approximately 300 μg per cigarette. Two of four individual studies of the pooled analysis indeed showed a statistically significant increase in nicotine-based acetone yields. No effect was seen in the pooled analysis for crotonaldehyde.

### 5.4. Summary, mainstream smoke chemistry

The mainstream smoke of research cigarettes manufactured without added sugar or with several levels of sugars applied as tobacco ingredients was quantitatively analyzed for constituents that have been considered to be of major toxicological relevance. The results of several studies of similar designs were pooled to increase the power of the statistical analyses in this review. In general, under ISO smoking conditions, a trend towards increasing nicotine-based yields of carbonyl constituents was observed, although with distinct differences among the particular carbonyl constituents. The most important increases were observed for formaldehyde and acrolein. A notable exception was acetaldehyde, which did not change with increasing sugar application levels. Furthermore, a decrease in the nicotine-based yields of several nitrogen-containing constituents was observed. The trends observed in this pooled analysis are in line with the findings of pyrolysis experiments. Similar trends were also observed if sugars were major parts of ingredient mixtures applied to research cigarettes. Sugar application-dependent effects were found to be less pronounced if smoke was generated under more intense smoking conditions. It should be considered that although the pooled analysis covers a list of 36 smoke constituents, which are considered representative of the major chemical classes of smoke constituents, it is only a small percentage of the number of approximately 5000 constituents currently known (Rodgman and Perfetti, 2009).

It is of note that all results in this section were based on the respective nicotine yield of the research cigarettes, which had to decrease with increasing sugar application levels. Thus, the nicotine-based yield data obtained in the individual or pooled analyses need to be interpreted in view of a trend to decreasing nicotine yields, which automatically could be considered an exaggeration of positive trends or an attenuation of negative trends with increasing sugar application levels compared to a more conventional way of reporting smoke constituent data on the basis of the number of cigarettes smoked or TPM or tar levels.

### 5.5. Toxicological interpretation of changes in mainstream composition

The pooled analysis revealed complex changes in smoke composition with, e.g. known human carcinogens increasing and decreasing in yield, which is difficult to interpret, in particular in view of the unclear etiology of smoking-related chronic diseases. Moreover, known hazards and risks of these compounds do in general not refer to them as constituents of a complex mixture but rather as neat compounds. Of the constituents identified as increasing with increasing sugar application levels, formaldehyde and benzene are classified as known human carcinogens, with predominant risks for nasopharyngeal cancer and hematopoietic malignancies, respectively (International Agency for Research on Cancer, 1982; International Agency for Research on Cancer, 2006). N-nitrosonornicotine and 4-aminobiphenyl, the yields of which were found to decrease with increasing sugar application levels, are also classified as human carcinogens (International Agency for Research on Cancer, 2007; Baan et al., 2008). N-Nitrosonornicotine can produce respiratory tract tumors in laboratory animals and has been classified as human carcinogen based on mechanistic considerations, whereas 4-aminobiphenyl increases the risk for bladder cancer.

A role of these smoke constituents in developing smoking-related cancer has been suggested but is unclear in terms of quantitative contributions and modes of action. For example, apart from the causal role for nasopharyngeal cancer, the International Agency for Research on Cancer argued that the overall balance of epidemiological evidence would not support a causal role of exposure to formaldehyde vapor for developing cancer of the oral cavity, oro-and hypopharynx, larynx, and lung (International Agency for Research on Cancer, 2006). Smoking has been associated with pharynx cancer (US Department of Health and Human Services, 2004), and thus, formaldehyde exposure from smoking may well contribute to this disease. Moreover, smokers are not exposed to just formaldehyde vapor but the whole complex matrix of smoke constituents, with a part of the smoke-borne formaldehyde residing in the particulate matter of smoke (Baker and Chang, 1999). This may change its potency and target organ specificity relative to pure formaldehyde inhalation. Using cancer extrapolation models from rats to humans (Conolly et al., 2004), it can be calculated that the daily average formaldehyde concentration a smoker would be exposed to would be around a “de minimis risk” of 10^−6^ for lifetime exposure depending on the model used, and this would also hold true for any additional formaldehyde exposure from using sugars as ingredients (Roemer et al., 2010). This estimate is much below the epidemiologically observed risk for respiratory tract cancer by smoking. However, this estimate is based on a model with large uncertainties (Crump et al., 2008), using one of several ways of assessing the exposure from smoking, and it may or may not mirror the actual role of formaldehyde in smoking-related carcinogenesis.

The proportion of smoking-induced total leukemia and acute myeloid leukemia attributable to the benzene taken up from cigarette smoke was assessed by combining epidemiologic data on the health effects of smoking with risk assessment techniques for low-dose extrapolation (Korte et al., 2000). The authors concluded that based on their modeling assumptions benzene would be estimated to be responsible for approximately 10–50% of smoking-induced total leukemia mortality. If this would indeed be the case, a further 9% increase at the maximum use level of sugars as ingredients could be meaningful.

Acrolein is the strongest irritant (Dorman et al., 2008) in the group of constituents with increased yields with increasing sugar application level. It has been suggested to play a role in smoking-related pathogeneses (Hecht, 2006; Rahman and Adcock, 2006) but, as for all other constituents, no quantitative attribution to the etiology of smoking-related diseases has been established. Thus, it is not obvious how to interpret these relative changes with increasing sugar application level in terms of the overall risks for cancer and non-malignant diseases for smokers.

Acrolein, benzene, formaldehyde, and N-nitrosonornicotine are also in the list of nine that was recommended for regulatory control of cigarette emissions (Burns et al., 2008; World Health Organization Study Group on Tobacco Product Regulation, 2008). 4-Aminobiphenyl is in the secondary list considered high priority for disclosure and monitoring.

The other smoke constituents found to increase with increasing sugar application level in the pooled analysis were 2-butanone, isoprene, toluene, and benzo[*k*]fluoranthene, while N-nitrosodimethylamine decreased. The latter is currently classified as probably carcinogenic to humans (International Agency for Research on Cancer, 1987), while isoprene, benzo[*k*] fluoranthene (and styrene, which was increased in the combined analysis of the HCI data) are classified as possibly carcinogenic to humans (International Agency for Research on Cancer, 1999; International Agency for Research on Cancer, 2002; International Agency for Research on Cancer, 2010). Toluene is not classifiable as to its carcinogenicity to humans, and 2-butanone is not listed at all. For non-cancer effects, toluene has been regulated based on neurological effects seen in humans (US Environmental Protection Agency, 2005), while 2-butanone has been regulated based on developmental toxicity (US Environmental Protection Agency, 2003). It is unclear whether any of the above toxicological properties of these constituents are indeed relevant for human smoking-related diseases.

Within the scope of the current assessment, the findings obtained in the chemical-analytical investigations of mainstream smoke were in the following section put into the broader perspective of biological toxicological tests utilizing the whole smoke or major fractions thereof, instead of concentrating on individual smoke constituents. In further sections, information was considered on smoke composition, exposures, and disease risks in major markets with predominant use of either American-blend or Virginia-type commercial cigarettes, which differ in their use of sugars as tobacco ingredients.

## 6. Effects of using sugars as ingredients in toxicological assays

The mainstream smoke of research cigarettes without or with various levels of sugars was investigated in *in vitro* cytotoxicity and genotoxicity assays, *in vivo* inhalation toxicity studies with primary emphasis on irritative changes in the respiratory tract, and in dermal tumorigenicity studies. The results of studies with similar design were pooled in analogy to the evaluation of the chemical composition.

### 6.1. *In vitro* cytotoxicity

**Figure 4:**
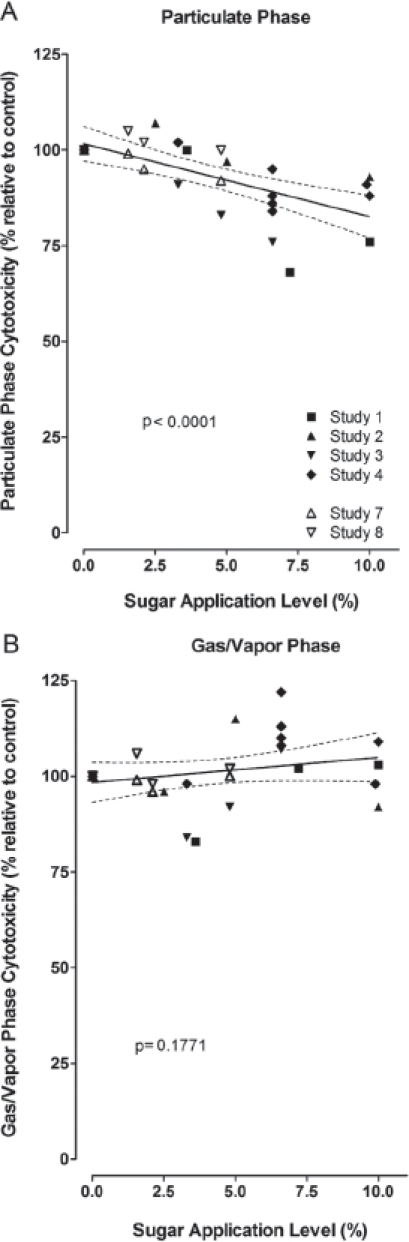
Cytotoxicity (EC_50_, TPM-based) of the mainstream smoke particulate and gas/vapor phases of research cigarettes with varying sugar application levels relative to the respective control. Linear regression with 95% confidence limits was performed. The legend in graph A also applies to graph B; for study references, see Table 3.

Cytotoxicity has been identified as a major toxicological activity of mainstream smoke and has been used for benchmarking comparisons (Roemer et al., 2004; Counts et al., 2006), the assessment of filter variations (Gaworski et al., 2009), and cigarette design changes (Tewes et al., 2003). In recent years, the Neutral Red Uptake assay (adapted from INVITTOX, 1990) has mostly been used to assess cytotoxic potential, and it is required for regulatory reporting purposes in Canada (Health Canada, 2000). Quite often, both the particulate and the gas/vapor phases have been tested separately. In order to improve the power of the current analysis, studies with applications of sugars as the sole ingredients (Roemer et al., 2010; Coggins et al., 2011) were evaluated by linear regression analysis. There was a statistically significant decrease in cytotoxicity activity for the particulate phase with increasing sugar application levels (Figure 4A, TPM-based comparison). This effect would not have been detected by evaluating these studies individually, except for study 3 using honey as an ingredient (Coggins et al., 2011). There was no change in cytotoxic activity for the gas/vapor phase (Figure 4B). The results of the current analysis are consistent with those studies, in which sugars were used as a major part of ingredient combinations at individual application levels up to 10.5% (Roemer et al., 2002; Baker et al., 2004a). In an additional study on the particulate fraction and the whole smoke of research cigarettes with HFCS at application levels of 3–5% (no control without sugar application), no difference in cytotoxicity was observed (Stavanja et al., 2006). No increase in *in vitro* cytotoxicity (ED_50_ per puff) or ciliatoxicity (effect after 4th puff) was seen in an early study on non-filter American-blend or single-blend Burley research cigarettes with and without 5.3% invert sugar (US Department of Health Education and Welfare, 1977).

### 6.2. *In vitro* genotoxicity

**Figure 5:**
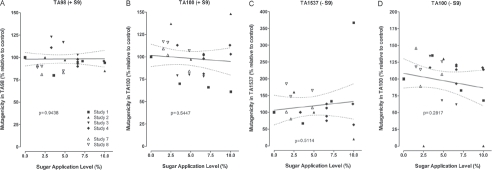
Bacterial mutagenicity (TPM-based) of the mainstream smoke particulate phase of research cigarettes with varying sugar application levels relative to the respective control. Selected conditions are tester strains TA98 and TA100 with metabolic activation (+S9) (A, B) and TA1537 and TA100 without metabolic activation (-S9) (C, D). Linear regression with 95% confidence limits was performed. The legend in graph A also applies to graphs B to D; for study references, see Table 3.

The mutagenicity of the particulate fraction of mainstream smoke has mostly been tested in the plate incorporation version of the *Salmonella typhimurium* reverse mutation assay, e.g. for the benchmarking comparison of commercial cigarettes (Roemer et al., 2004; Rickert et al., 2007b) or for the assessment of changes in cigarette filter design (Gaworski et al., 2009). This assay is required for regulatory reporting in Canada (Health Canada, 2000). For this type of investigations, the respective OECD guideline (Organization for Economic Co-operation and Development, 1997a) was adapted to the comparative testing of various types of smoke-derived test materials. The most sensitive tester strains are TA98 and TA100 in the presence of a metabolic activation system (S9). In pooling data sets on sucrose, invert sugar, honey, and HFCS from various studies and laboratories (Roemer et al., 2010; Coggins et al., 2011), mutagenicity data for these two testing conditions did not show any increase in activity with increasing single sugar application levels (TPM-based comparison, Figure 5A and B). No sugar application-dependent effect on mutagenicity was reported in any of the individual studies, including the results obtained with other tester strains and in the absence of metabolic activation (e.g. TA1537 with and TA100 without metabolic activation: Figure 5C and D). Various other studies used sugars in ingredient combinations at individual sugar levels up to 10.5%; no sugar-related effect on smoke mutagenicity was reported for any of these test cigarettes (Rodgman, 2002; Roemer et al., 2002; Baker et al., 2004a; Renne et al., 2006). In an additional study using condensate and whole smoke of research cigarettes with HFCS at application levels of 3–5% or with 3% HFCS replacing 3% corn syrup/invert sugar casings (no control without sugar application), no difference in bacterial mutagenicity was observed (Stavanja et al., 2006). Also, no difference was detected for these variations in sugar application in a sister chromatid exchange assay in Chinese hamster ovary cells. Similarly, comparing the use of 5% invert sugar versus that of 5% honey did not reveal any differences in response in genotoxicity assays (Stavanja et al., 2003). In an earlier study, in which cigarettes were soaked in solutions containing various types of sugars, also no increase in bacterial mutagenicity was observed for TA100 and rather a decrease for TA98 (Sato et al., 1979).

### 6.3. Subchronic inhalation toxicity

Subchronic inhalation toxicity studies in rats have become the most commonly used *in vivo* study type for the investigation of potential effects of changes in cigarette design on mainstream smoke toxicity (Terpstra et al., 2003), tobacco processing (Theophilus et al., 2003), or cigarette benchmarking comparisons (Patskan et al., 2008). Several studies were reported in which the effects of using sugars as tobacco ingredients on the toxicity of mainstream smoke were investigated. These studies were in general conducted in accordance with the respective guidance from OECD (Organization for Economic Co-operation and Development, 2009) with adaptation to the comparative investigation of smoke generated from different research cigarettes. During the 90-day inhalation period, clinical observations were conducted, body weight and food consumption were measured, and pulmonary function tests were performed. At scheduled necropsies, blood was collected for clinical pathology measurements, major organs were weighed, and tissues were collected for histopathological evaluation. The major emphasis in these studies was on potential effects in the respiratory tract. Some studies included a 42-day post-inhalation period to investigate the sustained development or recovery of effects.

The effects of sucrose and invert sugar, each at various application levels, were investigated in a large study, which shared the same control without sugar application (Table 4) (Coggins et al., 2011). At nominal TPM concentrations of 150 mg/m^3^, an increase in the formaldehyde concentration in the test atmospheres was observed that was dependent on the sugar application level. No such increase was found for acetaldehyde and acrolein. Analysis of exposure markers, such as blood cotinine or carboxyhemoglobin levels, as well as respiratory parameters, documented similar inhalation for the groups exposed to the smoke generated from the research cigarettes with the various levels of sugar applications. The usual smoke inhalation-related biological effects, such as a decreased gain in body weight and irritative changes in the respiratory tract epithelia, were observed. There was only one significant difference in histopathological findings between the groups exposed to the smoke generated from the research cigarettes with the highest sugar application level (10%) and that of the control without added sugars (Table 4): Increased incidence and severity score for respiratory epithelial hyperplasia at nasal level 2 was observed in male but not in female rats at the high sucrose application level. Significant differences were not observed for the low and medium sugar application levels (up to 7.2%). After a 42-day post-inhalation period, this effect had recovered. A few other findings were higher in rats previously exposed to smoke from cigarettes containing the highest sugar level than in those rats exposed to smoke from control cigarettes in one but not the other gender.

**Table 4:** Histopathological findings (mean severity scores) with significant differences between rats exposed to the smoke of research cigarettes with and without sugars applied as tobacco ingredients at the end of at least one subchronic rat inhalation study on mainstream smoke from research cigarettes with various application levels of sugars: sucrose and invert sugar (Coggins et al., 2011).

		Control	Sucrose			Invert sugar		
				
Finding	Gender	0%	3.6%	7.2%	10%	2.5%	5%	10%
Nose level 1,	M	2.3	2.0	1.9	2.6	2.3	2.8	2.8
goblet cell hyperplasia	F	1.0	0.8	0.5	1.3	1.1	1.6	0.7
Nose level 2,	M	0.7	0.8	1.0	**1.7**	1.3	1.1	1.6
respiratory epithelium hyperplasia	F	1.2	0.8	0.7	1.2	0.9	0.9	0.9
Nose level 2,	M	0.0	0.1	0.0	0.5	0.0	0.0	0.0
olfactory epithelium atrophy	F	0.7	0.8	0.4	1.6	0.3	0.6	1.0
Nose level 3,	M	0.0	0.0	0.0	0.0	0.0	0.0	0.0
olfactory epithelium atrophy	F	0.2	0.6	0.3	0.9	0.0	0.3	0.7
Larynx, arytenoid projections	M	2.3	1.8	1.9	2.2	2.1	2.1	2.2
squamous metaplasia	F	3.0	2.1	**1.4**	2.2	**1.8**	2.5	2.6

Severity scores on a scale of 0 to 4.

Additional groups not shown here: Air-exposed sham group as negative control, groups exposed to mainstream smoke of 1R4F standard reference cigarettes as quality control.

**Bold print:** significant effect compared to control (ANOVA followed by Dunnett's test, or if there was an increase of > 1.0 in the severity score).

The same laboratory conducted another large subchronic inhalation study with the same study design, in which research cigarettes with various application levels of HFCS alone (Coggins et al., 2011) or in combination with invert sugar and sucrose were investigated (Table 5). The concentrations of formaldehyde but not those of acet-aldehyde or acrolein in the test atmospheres increased with increasing sugar application levels. The only significant effects in this study were significantly higher goblet cell hyperplasia scores in the tracheas of female rats in groups exposed to the smoke of the test cigarettes with dual combinations of sugars at a total 6.6% application level. This effect was not seen with 6.6 or even 10% of HFCS alone or with the triple sugar combination at 9.9%, nor was it seen in the male rats. Few incidental effects were seen after the end of the post-inhalation period.

**Table 5:** Histopathological findings (mean severity scores) with significant differences between rats exposed to the smoke of research cigarettes with and without sugars applied as tobacco ingredients at the end of at least one subchronic rat inhalation study on mainstream smoke from research cigarettes with various application levels of sugars: high fructose corn syrup (HFCS), single (Coggins et al., 2011) and in combinations with sucrose and invert sugar.

		Control	HFCS			HFCS + Sucrose	HFCS + Invert Sugar	Sucrose + Invert Sugar	HFCS +Sucro + Invert Sugar
							
Finding	Gender	0%	3.3%	6.6%	10%	3.3 + 3.3%	3.3 + 3.3%	3.3 + 3.3%	3.3 + 3.3%+ 3.3%
Nose level 1,	M	2.2	1.2	1.9	2.2	1.6	1.4	1.8	1.6
goblet cell hyperplasia	F	1.4	0.9	0.8	1.5	0.7	0.8	1.0	0.2
Nose level 2,	M	2.1	1.7	2.4	2.2	1.6	1.8	2.0	1.8
respiratory epithelium hyperplasia	F	1.8	1.2	1.2	2.0	1.6	1.0	1.4	1.0
Nose level 2,	M	0.4	0.2	0.6	0.1	0.8	0.2	0.4	0.5
olfactory epithelium atrophy	F	1.0	0.3	1.6	1.2	1.7	0.4	0.9	1.1
Nose level 3,	M	0.0	0.0	0.0	0.0	0.5	0.2	0.5	0.0
olfactory epithelium atrophy	F	0.5	0.0	1.0	0.4	0.9	0.0	0.9	0.4
Larynx, arytenoid projections	M	3.8	3.5	3.8	3.7	3.7	3.9	3.7	3.7
squamous hyperplasia – metaplasia	F	3.7	3.9	3.6	3.6	3.7	3.7	3.8	3.9
Trachea,	M	2.6	2.1	2.8	1.9	3.2	2.7	3.3	3.3
goblet cell number/hyperplasia	F	2.3	2.6	3.1	2.6	3.7	3.7	3.4	1.7
Left lung,	M	3.2	2.5	3.3	2.6	3.4	3.2	3.3	3.5
goblet cell number/hyperplasia	F	3.5	3.0	3.2	3.6	3.9	3.6	4.0	2.4
Right lung,	M	2.2	2.0	1.8	1.6	2.7	2.0	2.3	2.3
goblet cell number/hyperplasia	F	2.7	3.0	2.3	3.1	3.4	2.6	3.3	1.9

Conditions as in Table 4, except of 2R4F as quality control.

A third subchronic inhalation study was conducted by the same laboratory and with the same study design using honey as the ingredient to be tested (Coggins et al., 2011). Again, formaldehyde concentrations in the test atmospheres were found to increase with increasing honey application levels in the test cigarettes, while there was no effect on the other aldehydes determined. The histopathological endpoints that showed significant sugar application-dependent differences in the above studies that tested sucrose, invert sugar, and HFCS were not different in this study (Table 6). However, a significant increase was seen for bronchial goblet cell hyperplasia in both males and females at the highest honey application level compared to control. No other significant differences were observed in the groups exposed to the smoke of the research cigarettes with honey, except of an increase in severity of olfactory epithelium atrophy at nose levels 2 and 3 of the male rats in the group exposed to the smoke of the research cigarettes with 6.6% honey. Such significant difference was not seen in female rats. There were no differences apparent after a 6-week post-inhalation period. No effect was seen at the 4.8% honey level, which is close to the maximum use level of sugar applications in most commercial cigarettes. Thus, in the current inhalation study, significant changes in groups exposed to smoke from research cigarettes with applied sugars vs. control groups were seen in endpoints again differing in comparison to the other two subchronic inhalation studies, and these changes had not been detected in the previous studies (Tables 4 and 5).

**Table 6:** Histopathological findings (mean severity scores) with significant differences between rats exposed to the smoke of research cigarettes with and without honey applied as tobacco ingredient at the end of at least one subchronic rat inhalation study on mainstream smoke from research cigarettes with various application levels of sugars (Coggins et al., 2011).

		Control	Honey		
			
Finding	Gender	0%	3.3%	4.8%	6.6%
Nose level 1,	M	2.1	1.8	2.6	1.8
goblet cell hyperplasia	F	1.5	1.3	1.7	1.5
Nose level 2,	M	2.3	1.8	2.4	2.2
respiratory epithelium hyperplasia	F	2.3	2.3	2.2	2.4
Nose level 2,	M	0.2	1.0	0.5	**1.9**
olfactory epithelium atrophy	F	1.0	1.3	1.0	1.7
Nose level 3,	M	0.0	0.5	0.2	**1.1**
olfactory epithelium atrophy	F	0.5	0.6	0.1	1.0
Larynx, arytenoid projections	M	3.6	3.7	3.6	3.3
squamous hyperplasia – metaplasia	F	3.5	3.1	3.1	3.6
Trachea,	M	2.0	1.3	2.9	2.4
goblet cell number/hyperplasia	F	1.3	1.6	1.1	1.2
Left lung,	M	2.2	1.9	3.0	2.8
goblet cell number/hyperplasia	F	2.6	2.3	2.6	2.8
Right lung,	M	1.5	1.6	2.0	**2.5**
goblet cell number/hyperplasia	F	1.4	1.7	1.7	**2.4**

Conditions as in Table 4, but with 1R4F and 2R4F as quality controls.

Although there were some significant differences in the groups exposed to the smoke generated from the research cigarettes with the highest sugar application levels, these were not consistently found when comparing between genders or between studies, suggesting that the few findings recorded were possibly due to chance rather than causally linked to the use of sugars as tobacco ingredients in these research cigarettes. If these differences would be triggered by the high sugar application level, one would expect to see very similar differences across those studies and across the sugars tested, based on the similarity of the changes determined in the comprehensive chemical-analytical studies on the mainstream smoke of these research cigarettes (see above). In any case, these differences were only observed at application levels higher than those usually applied to commercial cigarettes, although findings at exaggerated application levels may be indicative of effects at commercial application levels.

More light may be shed on this question by examining additional subchronic inhalation studies, in which research cigarettes with combinations of tobacco ingredients, including a major contribution of sugars, were evaluated. A study with similar design to the ones discussed above had sucrose and invert sugar incorporated into a an ingredient mixture with total sugar application levels of 3.3 and 5.1% (Vanscheeuwijck et al., 2002). Very similar smoke inhalation-dependent effects were seen, e.g. reduced body weight gain and histopathological findings in the respiratory tract. None of the differences discussed above for the sugar application studies was seen as a function of the ingredient mixture application. Among the multitude of endpoints investigated, there was only one significant increase observed for respiratory epithelium hyperplasia at nose level 1 in female but not male rats in the high sugar combination group. In another large study, brown and white sugar as well as invert sugar were used on research cigarettes as major part of different tobacco ingredient combinations at levels of 6.2, 10.5 and 7%, respectively (Baker et al., 2004a). The authors concluded that there were no discernible differences in the type or severity of treatment-related changes in the presence or absence of the ingredient combinations that included sucrose (as brown or white sugar) or invert sugar, since there were no statistically significant differences between rats exposed to the smoke from the control and test cigarettes in any of the 32 histopathological endpoints. There was also no difference in the histopathological findings in another subchronic rat inhalation study comparing research cigarettes with and without ingredient combinations, of which one included invert sugar at a final application level of 2% (Renne et al., 2006). These authors concluded that the presence of flavoring and casing ingredients did not significantly change the type or extent of toxicological effects observed in rat inhalation studies. As for some *in vitro* endpoints, no differences in response in subchronic inhalation studies in rats were observed if one type of sugar was replaced by another at the same application level (no controls without sugar application; Stavanja et al., 2003; Stavanja et al., 2006).

### 6.4. *In vivo* genotoxicity

As part of the above subchronic inhalation studies with single sugar ingredients (studies 1, 2, and 4 using sucrose, invert sugar, or high fructose corn syrup), the formation of micronuclei in rat bone marrow polychromatic eryth-rocytes and peripheral reticulocytes was investigated as an endpoint for *in vivo* clastogenicity (in basic accordance with Organization for Economic Co-operation and Development, 1997b). Peripheral reticulocytes were analyzed using a flow cytometric method capable of differentiating very young cells to overcome the splenic trapping of micronucleated cells which could have confounded this rat model (van Miert et al., 2008; Lynch et al., 2011). Inhalation exposure to mainstream smoke for up to 13 weeks did not increase the incidence rates of micronucleated reticulocytes in circulating blood, nor micronucleated erythrocytes in bone marrow compared with sham-exposed controls (data not shown). Although there were occasional significant differences between the groups exposed to smoke from cigarettes containing sucrose and invert sugar and those exposed to smoke from control cigarettes without added sugars, the overall conclusion was that the sugars had no impact on the formation of micronucleated red blood cells. Positive control samples responded in accordance to historical data from the same laboratory at all time points, indicating sensitivity of the rats to materials producing micro-nucleated cells.

### 6.5. Carcinogenicity studies

For a long time, mouse dermal carcinogenicity studies have been the only cancer bioassay available for cigarette smoke (reviewed by Coggins, 2007; Walaszek et al., 2007). In this assay, mainstream smoke condensate is chronically painted on the dorsal skin of mice, and skin tumors are the major biological response, which can be non-invasively examined on a daily basis. There are two principle experimental variants for this assay: Condensate is either applied to the skin during the whole experimental period as a test for complete carcinogenesis, or condensate is applied during the major experimental period of the study as a test for its promoting activity after initial treatment with a low dose of a known mouse dermal carcinogen, low enough not to lead to tumors on its own. There is one set of experiments, in which cigarettes with and without invert sugar were compared in a complete carcinogenesis model (US Department of Health Education and Welfare, 1977). The standard experimental blend in this study was made according to then non-filter American-blend cigarettes and included 5.3% invert sugar. It was compared to the same blend but without sugars. Groups of mice were treated with two doses of each condensate for 18 months. The major outcome of the study was the estimated probability of tumor avoidance and the estimated time until which 75% of the mice were without skin tumors based on life-table calculations. Statistical analysis revealed that the research cigarettes with and without invert sugar ranked very similarly for both dose groups, i.e. could not be differentiated (Table 7). If the tobacco humectant was omitted in one test cigarette, no difference in tumorigenic potential was seen in comparison to the reference cigarette containing the humectant. However, if both humectant and invert sugar were omitted, the condensate generated from the test cigarette had lower tumorigenic activity than that of the control (no explanation offered by the authors). In a parallel experiment, single-blend Burley cigarettes without and with sugar addition were compared (twice at the same dose) and no difference in tumorigenic response of the condensates was observed (Table 5). Notably, in the same study the condensates of Burley and American-blend cigarettes could well be differentiated. Taken together, these experiments suggest that sugar by itself had no impact on the tumorigenicity of smoke condensate in the mouse dermal carcinogenicity assay.

**Table 7:** Dermal tumorigenicity of mainstream smoke condensate generated from research cigarettes with and without invert sugar as tobacco ingredient.

		Estimated probability of tumor avoidance (M ± SE)		Estimated number of days until 75% of mice remained free of tumors	
			
Research cigarette type	Condensate dose group	No invert sugar	With invert sugar	No invert sugar	With invert sugar
Standard experimental blend III	12.5 mg	0.715±0.053	0.711±0.026	529	515
	25 mg	0.432 ±0.060	0.449 ±0.028	376	414
Burley blend*	12.5 mg	0.508 ±0.059	0.586 ±0.056	420	452
	12.5 mg	0.532	0.509	417	372

Life-table-corrected data (US Department of Health Education and Welfare, 1977) (no sugar-dependent differences for formaldehyde, acetaldehyde, acrolein, and tar yields).

* Two times low dose; Burley blend and standard experimental blend III tumor probability data (low dose) were statistically significantly different.

No other carcinogenicity study is available comparing cigarettes with appreciably different levels of sugars. In a study to evaluate the tumorigenic activity of research cigarettes without and with a combination of flavors, the test cigarettes had additional glucose and honey at 0.03% each on a background of 2% brown invert sugar syrup in both the test and control cigarettes; no difference in tumor promoting activity was found between test and control cigarette condensates (Gaworski et al., 1999). In two other dermal tumor promotion studies, one type of sugar was replaced by another at the same application level, and no differences in tumor promoting activity could be observed in terms of tumor incidences or multiplicities (5% invert sugar by 5% honey: Stavanja et al., 2003; 3% corn syrup/invert sugar by 3% HFCS: Stavanja et al., 2006).

## 6.6. Summary, toxicological assays

Complimentary to the chemical-analytical investigations, *in vitro* cytotoxicity, genotoxicity, subchronic inhalation, and carcinogenicity studies have been available for the non-clinical assessment of the toxicological effects of using sugars as ingredients. At sugar application levels up to 10%, which is twice the usual maximum application level, no consistent toxicologically relevant changes in the activity in these assays were observed; statistically significant changes seen in one inhalation study could not be confirmed in others using similar types of sugars and study designs. This is particularly important in view of the statistically significant increases in nicotine-based yields of acrolein and formaldehyde observed in smoke from cigarettes with increasing sugar application level. Formaldehyde and in particular acrolein play relatively prominent roles in the *in vitro* cytotoxicity assay (Tewes et al., 2003) and can elicit irritative effects in the upper respiratory tract in rat subchronic inhalation studies (Feron et al., 1978; Woutersen et al., 1987). The acrolein concentrations in the above described smoke inhalation studies (< 1.1 mg/m^3^) (Coggins et al., 2011) were similar to the lowest observed effect levels in this subchronic model for histopathological changes in the nose, the lateral wall respiratory epithelium being the most sensitive site (Dorman et al., 2008). At higher acrolein concentrations, mucus hypersecretion in the airways of rats was stimulated (Borchers et al., 1998). The formaldehyde concentrations in the smoke inhalation studies (<0.5mg/m^3^) were below the lowest observed effect level observed in the subchronic formaldehyde inhalation study (Woutersen et al., 1987) and below thresholds considered to be relevant for genotoxic and carcinogenic effects (Nielsen and Wolkoff, 2010). At no-toxic effect levels, neither additive nor potentiating effects occurred from the combined exposure to these aldehydes (Cassee et al., 1996). In principle, the lack of additive or potentiating effects in defined mixtures of aldehydes does not exclude an additive or potentiating effect by the increased aldehyde levels on the background of smoke-related findings seen in these inhalation studies, but such effect was not observed. Thus, the totality of the reviewed studies suggests that the increased aldehyde yields determined in the chemical analyses did not increase the activity of the smoke in toxicological assays that are in principle sensitive to their activity. Moreover, the overall changes by the use of sugars as ingredients determined in the chemical analyses may either have balanced each other out or were not large enough to significantly affect the toxicological activity that is inherent to cigarette smoke in the assays employed.

## 7. Comparison of marketed American-blend and Virginia-type cigarettes

The above non-clinical studies should ideally be complimented by clinical and epidemiological data on exposure and effects differentiating between smokers smoking cigarette types with and without sugars used as tobacco ingredients. However, such data for a direct comparison of commercial cigarette types that differ by only this one parameter are not available, with the exception of menthol versus non-menthol cigarettes (Werley et al., 2006; Heck, 2010). As a substitute, we compared data from markets of primarily American-blend and Virginia-type cigarette consumption, respectively. These cigarette types differ in their blend composition, but also in their natural sugar content and the level of sugar applied as ingredient.

### 7.1. Relative impact of sugar application on chemical composition of mainstream smoke in marketed American-blend and Virginia-type cigarettes

The chemical composition of mainstream smoke generated from American-blend and Virginia-type cigarettes is qualitatively similar, with some quantitative differences, most of which can be explained by the differences in tobacco blend composition. For instance, the higher yields of nitrogen-containing smoke constituents observed for single-blend Burley compared with single-blend Virginia research cigarettes (Adam et al., 2006; Ding et al., 2008) carries over to American-blend compared to Virginia-type cigarettes, in particular resulting in higher yields of most of the tobacco-specific N-nitrosamines, nitrogen oxides, ammonia, and aromatic amines in American-blend cigarettes (Gregg et al., 2004; Counts et al., 2005; Hyodo et al., 2007; Hammond and O'Connor, 2008). High molecular weight polycyclic aromatic hydrocarbons, such as benzo[*a*]pyrene and benzo[*k*]fluoranthene, are formed at lower yields in the mainstream smoke of single-blend Burley than in that of Virginia research cigarettes (Ding et al., 2008), which also carries through to mostly lower yields in American-blend compared to Virginia-type market cigarettes (Ding et al., 2006). This difference has been related to the higher nitrate content in Burley-containing cigarettes, which may interfere with the pyrolytic formation of polycyclic aromatic hydrocarbons. Formaldehyde yields are generally lower in the smoke of American-blend than in that of Virginia-type cigarettes, which is considered to be related to the lower overall sugar content and higher levels of nitrogen-containing compounds (Leffingwell, 1999) in American-blend compared to Virginia-type cigarettes.

The pooled chemical analyses of the mainstream smoke of research cigarettes as described above suggested that there are statistically significant increases in the slopes of the linear regressions in the nicotine-based yields of seven smoke constituents with increasing sugar application levels, most importantly for formaldehyde, benzene, and acrolein. The increased yields were mainly observed using research cigarettes with sugar application levels beyond the maximum application level of 5% used in commercial cigarettes. Consequently, marketed American-blend cigarettes, which do not have a higher total sugar content than marketed Virginia-type cigarette, do not show an increased yield of these smoke constituents in direct comparisons (Table 8). Rather, the nicotine-based yield of formaldehyde from American-blend cigarettes is on average 20–40% lower than that from Virginia-type cigarettes, independent of the machine-smoking condition used for the smoke generation. For acrolein, 2-butanone, benzene, and toluene, no relevant differences in nicotine-based yields were found when comparing American-blend and Virginia-type market cigarettes. Benzo[*k*]fluoranthene, the one polycyclic aromatic hydrocarbon with statistically significant increased yield in the pooled analysis, has not been determined in market benchmarking studies with one exception, in which on average 27% lower yields were determined for American-blend compared to Virginia-type cigarettes (Ding et al., 2006). This agrees with the findings of numerically lower nicotine-based yields for benzo[*a*] pyrene, which is mostly determined as the only representative of this class of constituents, for American-blend versus Virginia-type market cigarettes (Table 8). Thus, the use of sugars as ingredients may not cause any concerns regarding this class of smoke constituents. As mentioned above, the largest difference in the composition of the smoke generated from American-blend or Virginia-type cigarettes stems from nitrogen-containing compounds (Table 6). Of these, nicotine-based yields of N-nitrosodimethylamine, N-nitrosonornicotine, and 4-aminobiphenyl were found to decrease in a statistically significant manner with increasing sugar application levels in the pooled analysis. Taken together, those constituents found to significantly increase in research cigarettes with increasing sugar application levels were similar or even lower in American-blend than in Virginia-type market cigarettes, whereas the use of sugars as tobacco ingredients in American-blend cigarettes attenuates the yields of some of those constituents which are high in American-blend compared to Virginia-type market cigarettes.

**Table 8:** Selected mainstream smoke constituent yields (per mg nicotine) for marketed American-blend and Virginia-type cigarettes (Counts et al., 2005; Gregg et al., 2004; Hammond and O'Connor, 2008).

	Yields (M ± SD) (mg nicotine)-^1^						
		
	ISO smoking conditions			HCI smoking conditions			
			
Constituent	American-blend	Virginia-type	Difference§ (%)	American-blend	Virginia-type	Difference§ (%)	Reference
Formaldehyde (μg)	45	81	−44	46	76	−39	Hammond
	31±12	40 ±14	−21	39 ±13	68 ±23	−42	Counts
	28 ±12	34 ±16	−18				Gregg
Acetaldehyde (μg)	641	656	−2	615	513	**+** 20	Hammond
	578 ±122	589±111	−2	686 ±163	652 ±159	+ 5	Counts
	673 ±123	820 ±123	−18				Gregg
Acrolein (μg)	63	80	−21	65	67	−3	Hammond
	52 ±12	53±5	−2	68±16	67 ±18	+ 0	Counts
	58±11	68±16	−15				Gregg
2-Butanone (μg)	68±15	74 ±17	−8	92±21	93 ±19	−1	Counts
	74 ±14	95 ±14	−22				Gregg
Benzene (μg)	47	51	−8	41	37	+ 11	Hammond
	51±10	51±17	+ 0	39±8	39±5	+ 0	Counts
	52±9	58±8	−10				Gregg
Toluene (μg)	76	78	−3	74	65	+ 14	Hammond
	74 ±14	71±18	+ 4	72 ±15	65±9	+ 10	Counts
	85 ±14	92 ±16	−7				Gregg
Benzo[*a*]pyrene (μg)	7.9	13.1	−40	7.3	10.0	−27	Hammond
	11.5±3.0	10.8±1.2	+ 6	8.8±2.2	10.3±2.7	−15	Counts
	12.0±1.0	15.8±1.8	−24				Gregg
N-Nitrosonornicotine (ng)	187	28	+567	161	22	+632	Hammond
	149 ±78	25±6	**+496**	108±47	17±0	**+538**	Counts
	94 ±16	53 ±34	**+ 76**				Gregg
4-Aminobiphenyl (ng)	2.4	1.9	+ 26	2.1	1.5	**+** 40	Hammond
	3.1±0.8	2.1±0.9	**+ 50**	2.2 ±0.5	1.3±0.1	**+ 73**	Counts
	1.4 ±0.3	1.2 ±0.4	+ 17				Gregg

Smoking conditions: ISO: International Standardization Organization; HCI: Health Canada Intense.

§ Difference of nicotine-based yields of American-blend vs. Virginia-type cigarettes;

**Bold print:** statistically significant difference between both types of cigarettes (not available for Hammond dataset for nicotine-based yields).

In summary, in American-blend compared to Virginia-type cigarettes, parts of the Virginia tobacco are replaced by Burley tobacco, which is practically free of sugars. The resulting decrease in the natural sugar content in the blend is partly replenished by applying sugars as ingredients to levels at or below that found in Virginia-type cigarettes. Thus, the sugar-dependent findings in the pooled analysis of smoke constituent yields from research cigarettes do not carry over to increased yields of these constituents in the blended cigarettes compared to Virginia-type cigarettes. For instance, the observed increases in formaldehyde, acrolein, or benzene by sugar application to research cigarettes do not result in higher average yields of these constituents in American-blend compared to Virginia-type cigarettes. On the contrary, formaldehyde yields tend to be higher on average in Virginia-type cigarettes. The sugar-dependent decreased yields of some nitrogen-containing smoke constituents seen in the pooled analysis of the research cigarettes alleviate the naturally higher yields of these constituents in the smoke from Burley-containing cigarettes.

### 7.2. Comparison of marketed American-blend and Virginia-type cigarettes in toxicological assays

The comparative activity of American-blend and Virginia-type cigarettes was assessed in *in vitro* cytotoxicity and genotoxicity assays, mostly by using the mainstream smoke of reference cigarettes representative of the two types of market cigarettes. Smoke from Virginia-type cigarettes was consistently more cytotoxic than that of American-blend cigarettes (Bombick et al., 1998; Roemer et al., 2004; Rickert et al., 2007a). This was true for the particulate and the gas/vapor phase, which were investigated in two of the studies. These results are consistent with the tendency to higher cytotoxicity of Virginia compared to Burley single-blend research cigarettes (Pickett et al., 2010), while in a larger inter-laboratory study on the cytotoxicity of the particulate phase on average no such differences were found (Pickett et al., 2010).

In the Salmonella reverse mutation assay, mainstream smoke condensate from American-blend cigarettes was consistently more mutagenic than the condensate of Virginia-type cigarettes (Mizusaki et al., 1977; Roemer et al., 2004; Rickert et al., 2007a). This result is consistent with the higher mutagenicity of smoke from Burley tobacco compared with that from Virginia tobacco (Mizusaki et al., 1977; Yoshida and Matsumoto, 1980; Gairola, 1982; Clapp et al., 1999; DeMarini et al., 2008). In mammalian cell-based genotoxicity assays, in contrast to the bacterial assay and depending on the specific assay type used (and on the laboratory conducting the assay), particulate matter from single-blend Burley mostly tended to be less active than that from Virginia cigarettes (Schramke et al., 2006; DeMarini et al., 2008).

In subchronic inhalation studies, the smoke from Virginia-type cigarettes seemed to have higher potency than that of single-blend Burley or American-blend cigarettes regarding histopathological changes in the larynx (compiled by Smith, 1990). Histopathological findings in the trachea and lungs were of similar degree. Dermal carcinogenicity studies in mice were not able to clearly differentiate between the condensates generated from American-blend and single-blend Virginia and Burley research cigarettes. If anything, there was a trend towards slightly higher tumorigenic potency for Virginia than Burley condensate, with that of the American-blend cigarettes being somewhat intermediate (Wynder and Hoffmann, 1963; Dontenwill et al., 1976; US Department of Health Education and Welfare, 1980).

The comparison of the market cigarette types indicates that the differences in toxicity observed between the smoke of Virginia-type and American-blend cigarettes appear to reflect the toxicity of the tobacco blend components rather than the application of sugar.

Current scientific knowledge does not allow a conclusion about which of these assay types or particular endpoints are more predictive of the induction of smoking-related diseases in humans. In summary, the results of the toxicological assays on research and market cigarettes confirm that cigarette smoke is toxic; however, they also suggest that the use of sugars applied to the tobacco of American-blend cigarettes at current use levels is unlikely to increase the inherent toxicity of tobacco smoke.

### 7.3. Simulated uptake in smokers of mainstream smoke constituents at maximum sugar application level and from marketed American-blend and Virginia-type cigarettes

In order to further evaluate the changes in mainstream smoke chemistry that were determined in the pooled analysis described above as a consequence of using sugars as cigarette tobacco ingredients, a simulation of the differential smoking-related exposure to those constituents was performed, for which statistically significant quantitative changes were identified. This approach offers the most discriminatory analysis of potential changes in mainstream smoke exposure resulting from the use of a particular ingredient in a research cigarette, but it has some limitations with regard to the extrapolation of the results to market cigarettes and their actual use by consumers. Therefore, the hypothetical changes in exposure to particular constituents from research cigarettes at 0 and 5% were simulated and compared to those from average American-blend and Virginia-type market cigarettes.

**Figure 6:**
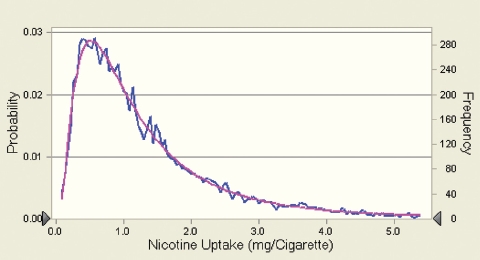
Monte Carlo simulation of the nicotine uptake distribution based on nicotine equivalents from a population-based biomonitoring study with smokers of American-blend cigarettes. Data from (Scherer et al., 2007), corrected for 85% recovery of nicotine and its metabolites; log-normal Monte Carlo simulation; blue line: forecast values; magenta line: fitted line (mode: 0.52mg/cig.; median: 1.03mg/cig.).

Most information on exposure and uptake of cigarette smoke constituents has been generated for nicotine via the determination of nicotine and some of its major metabolites in human body fluids, such as saliva, plasma, or urine. If trans-3'-hydroxycotinine, cotinine, and nicotine are determined together with their glucuronide conjugates, approximately 80–95% of the nicotine uptake may robustly be determined in terms of “nicotine equivalents,” eliminating much of the effect of inter-individual metabolic differences (Tricker, 2006). Assuming proportional relationships between the yields of nicotine and most other constituents (Roemer et al., 2004; Counts et al., 2005; Hyodo et al., 2007; King et al., 2007), the uptake of these constituents and their distribution among individual smokers can be predicted by bridging from known nicotine uptake distributions. Such uptake distributions offer a more informed estimate of the impact of potential changes in smoke composition than just point estimates for nicotine uptake. Using Monte Carlo simulation, the mean and standard deviation of nicotine equivalents determined in a German population-based biomonitoring study with American-blend cigarettes (Scherer et al., 2007) was connected with the mean and standard deviation of the nicotine-based yields for the respective constituents at the 0 or 5% sugar addition levels, respectively, as determined in the pooled analysis. This approach enables calculation of uptake distributions per cigarette which provide insight beyond the simple results of standard machine-smoking conditions and incorporates the variability of human smoking behavior as best as possible. This variability is reflected in the broad distribution of the uptake of nicotine equivalents after Monte Carlo simulation (Figure 6) and propagates to the distribution simulated for any of the other constituents. A log-normal model was used for the simulation, which seems to be justified in view of more recent and more detailed distribution data on nicotine uptake (Mendes et al., 2009), although higher modes for the distributions were obtained than in the current simulation. The same simulation was applied to the average nicotine-based yields of smoke constituents of the market cigarettes.

**Figure 7:**
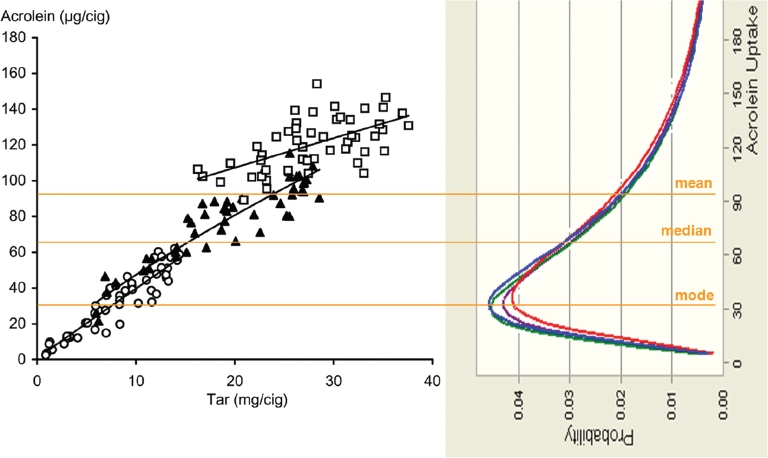
Simulation of acrolein uptake distributions for research cigarettes with 0 and 5% sugar application and American-blend and Virginia-type market cigarettes (right panel) in comparison to acrolein yields obtained by three machine-smoking conditions (left panel, reproduced with permission; Counts et al., 2005). Machine-smoking data compared to the fitted log-normal Monte Carlo simulation of acrolein uptake using Crystal Ball; left panel: ○: ISO smoking conditions (International Organization for Standardization, 1991), ▴: Massachusetts smoking conditions (Massachusetts General Laws Annotated, 1997), □: Health Canada smoking conditions (Health Canada, 2000); right panel: blue line: research cigarettes with 0% sugar addition; red line: research cigarettes with 5% sugar application; green line: American-blend market cigarettes, violet line: Virginia-type market cigarettes.

The distributions calculated for acrolein uptake from smoking hypothetical research cigarettes at 0 and 5% sugar application level as well as that from the average yield data of American-blend and Virginia-type cigarettes almost completely overlap (94% of the distribution at 5% sugar application level coincides with the distribution at no sugar application; Figure 7). Nevertheless, the modes of these distribution curves reflect the small differences in nicotine-based acrolein yields calculated from machine-smoking of the respective research and market cigarettes (Tables 3 and 8), i.e. a slight increase from 0 to 5% sugar application level but a smaller simulated uptake from American-blend vs. Virginia-type market cigarettes. For the validation of this approach, the simulations for acrolein uptake were compared with the acrolein yields of market cigarettes (Counts et al., 2005) if machine-smoked at three standardized conditions (International Organization for Standardization, 1991; Massachusetts General Laws Annotated, 1997; Health Canada, 2000). This is possible since practically all nicotine and acrolein inhaled from smoking are absorbed (Baker and Dixon, 2006). The median acrolein uptake per cigarette determined by the simulation is similar to the average yield obtained when this selection of cigarettes was smoked under intermediately intense conditions (Massachusetts General Laws Annotated, 1997). This similarity is reasonable as in a recent review on actual human smoking conditions (Bernstein, 2004), median values for interpuff interval, puff duration, and puff volume of 28 s, 1.9 s, and 43 ml, respectively, were found to be very close to the respective values under these intermediate machine-smoking conditions (30 s, 2 s, and 45 ml).

**Figure 8:**
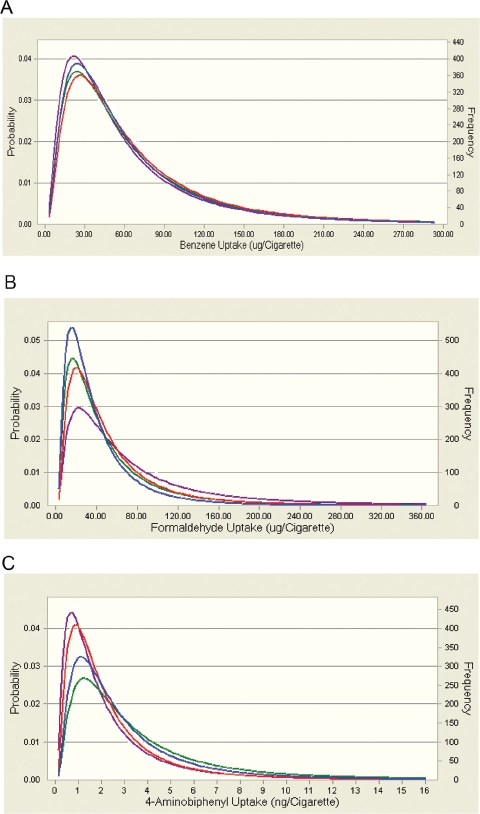
Simulation of benzene (A), formaldehyde (B), and 4-aminobiphenyl (C) uptake distributions for research cigarettes with 0 and 5% sugar application and American-blend and Virginia-type market cigarettes. For color legend, see explanations for the right panel in Figure 7.

The uptake distributions for benzene (Figure 8A), toluene, benzo[*k*]fluoranthene, and NDMA also overlapped to a very large degree indicating no relevant difference by sugar application and market type cigarette. Uptake distributions per cigarette are also shown for formaldehyde (Figure 8B) and 4-aminobiphenyl (Figure 8C) representing smoke constituents with the largest percentage increases or decreases in nicotine-based yields with increasing sugar application levels. The hypothetical formaldehyde uptake increased by sugar application to the research cigarettes (overlap of 86%), but this increase was still smaller than the increase from American-blend to Virginia-type market cigarettes. The hypothetical uptake of 4-aminobiphenyl was higher for American-blend than for Virginia-type cigarettes, while it got smaller with sugar application. Thus, the uptake simulations reflect the changes in the point values determined in the pooled analysis of the research cigarettes or the differences in the average yield data of the market cigarettes, but these changes and differences are put into the perspective of the broad inter-individual variability in the uptake of these constituents by smokers.

In a recent benchmark study for biomarkers of exposure to smoke constituents, groups of smokers from Germany and the UK as prototypic markets of American-blend and Virginia-type cigarettes were compared (Lindner et al., 2011). Smoking-attributable excretion levels of 3'-hydroxypropyl-mercapturic acid, a urinary acrolein-related biomarker of exposure, were 1.19 and 1.13 mg per 24 h for German and the UK smokers, respectively, with relative standard deviations of approximately 100% (and on a nonsmoking background of approximately one third of the smokers' levels). For S-phenylmercapturic acid, a marker for benzene exposure, urinary marker levels were 3.8 and 3.6 μg per 24 h German and UK smokers, respectively, while those for 4-aminobiphenyl were 26 and 21 ng per 24 h. Thus, the biomarker data confirm the similarity of exposures to these constituents on the basis of means and standard deviations (as a measure of variance) by the two types of cigarettes as suggested by the current modeling approach based on analytical-chemical data. The authors concluded that the contribution of the country and thus the type of cigarettes smoked to the total variation was marginal for all biomarkers of exposure investigated (Lindner et al., 2011).

### 7.4. Comparison of American-blend and Virginia-type markets regarding smoking behavior

The above approach of bridging from the uptake of nicotine to that of other smoke constituents is only reasonable as a determinant of potential changes of exposure by the use of sugars as ingredients, if there is no change in smoking behavior to be expected. There are no studies available that have assessed the potential direct effect of using sugars as ingredients on smoking behavior. However, studies on “exotically” flavored cigarettes did not reveal any difference in smoking behavior compared to regular American-blend cigarettes (O'Connor et al., 2007), nor have there been differences in exposures between smokers of mentholated and non-mentholated cigarettes (Heck, 2009; Wang et al., 2010). Thus, it is not expected that there would be any difference in overall smoke exposure from using sugars as ingredients.

**Figure 9:**
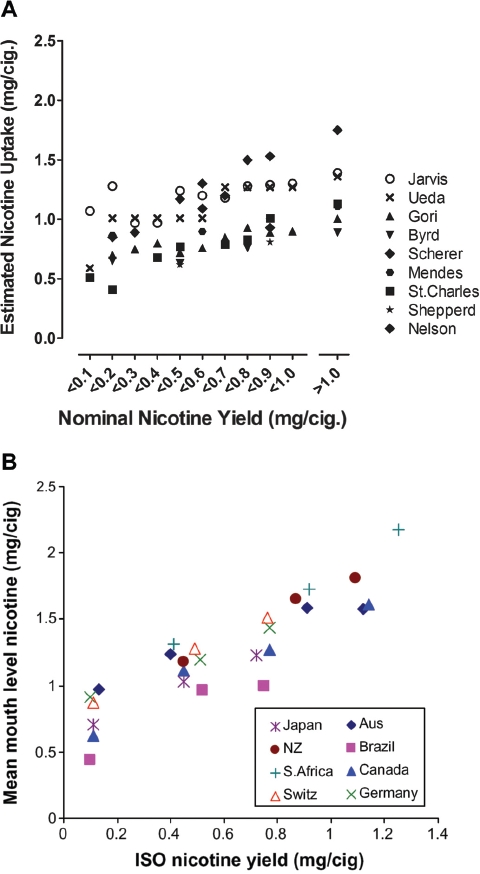
Survey of nicotine uptake estimates per cigarette in smokers (mg/cig.). A: Estimated nicotine uptake from plasma, urine (based on nicotine equivalents for plasma and urine), or cigarette filter analyses (mouth level exposure). Cigarettes of American-blend (Byrd et al., 1998; Gori and Lynch, 1985; Mendes et al., 2009; Scherer et al., 2007; Shepperd et al., 2009; St Charles et al., 2010), Virginia-type (Jarvis et al., 2001), or unknown nature (Ueda et al., 2002) were smoked in these studies. B: Estimated nicotine uptake determined as “mouth level exposure” based on cigarette filter analyses in eight different countries with varying blend preferences (reproduced with permission; Mariner et al., 2011).

This interpretation is further corroborated by the lack of differences for nicotine uptake per cigarette from smokers from various regions of the world including those of predominantly American-blend and Virginia-type markets, which among other parameters differ by the use of sugars as ingredients (Figure 9A). The intake of nicotine per cigarette was also considered to be similar in a study that included smokers from Brazil, Mexico, China, and Poland (Blackford et al., 2006). A most recent international comparison determined nicotine exposure (“mouth level exposure,” MLE) based on nicotine residues in the filters of smoked cigarettes collected in eight countries (Mariner et al., 2011). No differences between those countries for MLE in dependence of the ISO nicotine yield of the cigarettes smoked were seen (Figure 9B). However, when the cigarettes across the countries were separated into Virginia- and American-blend types, overall MLEs for smokers of Virginia-type cigarettes were significantly greater than those of American-blend smokers, reflecting differences (and preferences) in ISO yields. After adjusting the MLE data for the differences in ISO yields, the nicotine (and tar) MLEs per cigarette were higher for the American-blend than the Virginia-type smokers. Thus, no clear trend that could be related to the use of sugars as ingredients could be observed either. Such an ecological approach of comparing markets has also been chosen by the International Tobacco Control Policy Evaluation Survey (Hammond et al., 2004) and others (International Agency for Research on Cancer, 2007; European Commission Health and Consumer Protection Directorate-General Scientific Committee on Emerging and Newly Identified Health Risks, 2008) in order to deduce potential determining principles and trends in tobacco exposure and related diseases from the comparison of national data.

**Figure 10:**
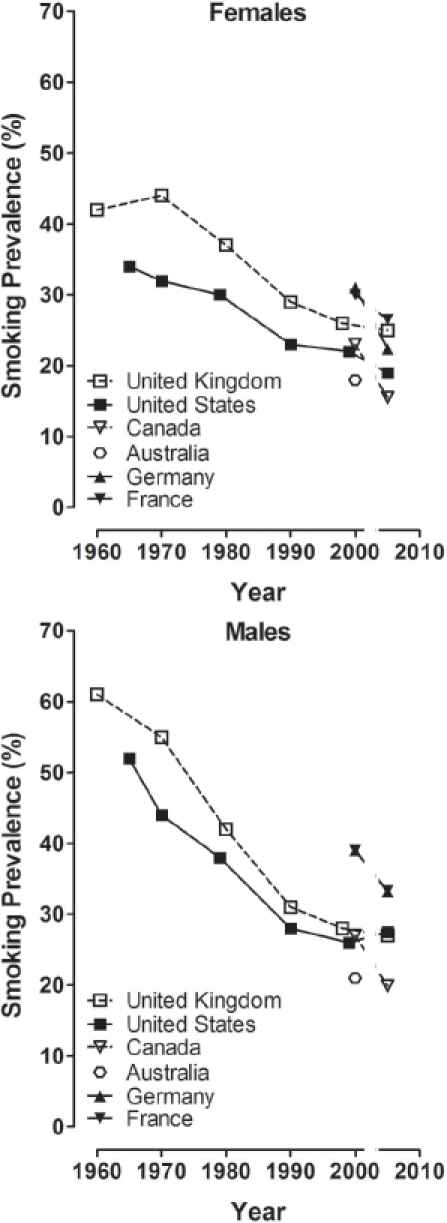
Smoking prevalences in American-blend and Virginia-type cigarette markets. Data from American-blend (United States, Germany, and France) and Virginia-type markets (United Kingdom, Australia, and Canada) (MacKay and Eriksen, 2002; World Health Organization, 2008: data from approximately 2005).

Further variables of smoking behavior in a population are smoking prevalence and intensity. Smoking prevalence data have been published by many national and international sources. Data published by the World Health Organization demonstrate wide international differences in smoking prevalence (MacKay and Eriksen, 2002; World Health Organization, 2008), but do not indicate trends by the predominant type of cigarette smoked in a particular country (Figure 10). In an extensive study comparing exposure and chronic disease risk between countries with predominant American-blend (United States, Germany, Austria, and Denmark) and countries with predominant Virginia-type smokers (United Kingdom, Australia, and Canada), stratified by age group, gender, and time periods from 1971 to 2000, no overall statistically significant difference in smoking prevalence could be detected (Lee et al., 2009). The ex-smoking prevalence was generally increasing over the time periods investigated in both sexes and all age groups and was lower in American-blend than Virginia-type markets, although only rarely with statistical significance. The International Tobacco Control (ITC) Four Country Survey investigated quit rates and cessation in Australia, Canada, the United Kingdom, and the United States (Hyland et al., 2006). They concluded that predictors of making quit attempts and cessation were similar for each of the four countries. Although there were some differences among the countries in predictors of success, such as heaviness of smoking index, favorable attitudes about smoking, and self-efficacy, these differences could not be associated with American-blend or Virginia-type preferences in these countries.

**Figure 11:**
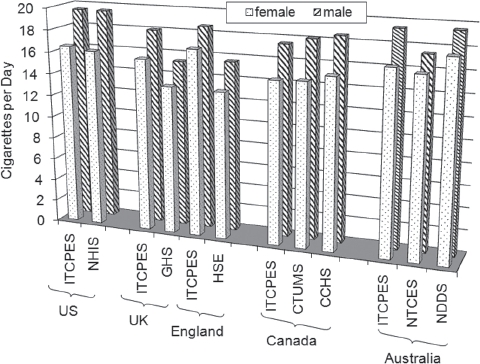
Daily cigarette consumption in predominantly American-blend (United States) and Virginia-type (United Kingdom/England, Canada, Australia) markets for male (hatched bars) and female (dotted bars) smokers. Data of the years 2000-2002 taken from International Tobacco Control Policy Evaluation Survey (Hammond et al., 2004): ITCPES: International Tobacco Control Policy Evaluation Survey; NHIS: National Health Interview Survey; GHS: General Household Survey; HSE: Health Survey for England; CTUMS: Canadian Tobacco Use Monitoring Survey; CCHS: Canadian Community Health Survey; NTCES: National Tobacco Survey Evaluation Campaign; NDDS: National Drug Strategy Household Survey.

The most frequent measure of smoking intensity is the number of cigarettes smoked per day. For this variable over all time periods and age groups, no difference was observed between American-blend and Virginia-type markets, while smoking intensity was higher in males than in females (approximately 20 vs. 16 cigarettes per day) (Lee et al., 2009). This gender difference in smoking intensity and the lack of difference between American-blend and Virginia-type markets (Figure 11) can also be derived from data from the International Tobacco Control Policy Evaluation Survey (Hammond et al., 2004; Hyland et al., 2006). In an international comparison, England as a predominantly Virginia-type market had intermediate scores in the Fagerström Test for Nicotine Dependence, while Germany and the United States as predominantly American-blend markets showed the most extreme differences (Fagerström and Furberg, 2008). Those scores were inversely related to smoking prevalence rates in these countries, which again puts the Virginia-type market into the range observed for American-blend markets. Likewise, no distinction could be made for a number of smoking dependence-related parameters, including smoking intensity, heaviness of smoking index, and number and duration of quit attempts, between smokers from American-blend and Virginia-type markets (Shahab et al., 2008).

Summarizing the data detailed above, there are no indications that the use of sugars as ingredients, which is one characteristic of American-blend cigarettes, has any influence on smoking behavior and behavior-related exposure, either in terms of cigarette smoking prevalence, the frequency of quit attempts, smoking intensity as cigarettes per day, and nicotine uptake. In addition, these data do not in any case support the suggestion that the use of sugars as ingredients or any other ingredients in American-blend cigarettes would lead to an increased dependence potency by whatever mechanism hypothesized.

### 7.5. Comparison of American-blend and Virginia-type markets regarding smoking-related disease risks

Predominantly American-blend and Virginia-type markets were compared for the risks of mortality from smoking-related lung cancer and COPD (Lee et al., 2009), two major serious diseases that can be caused by chronic smoking. Unadjusted mortality rates were generally lower early on in markets with predominant Virginia-type cigarettes, with the difference diminishing or reversing by the 1990s. Differences by cigarette type were rarely significant for age and time period groupings, due to variations, particularly for COPD, between countries within cigarette type. Conclusions based on estimated smoking-related excess mortality were similar to those based on unadjusted mortality rates: There was little indication of any difference between American-blend and Virginia-type cigarettes on risk of lung cancer or COPD. The approach of this study had of course less discriminative power than any analytical-chemical study, which is partly due to unspecified differences between countries with the same type of predominant cigarette type. It was estimated that this study could have detected differences of 25–40% for male lung cancer, or twofold differences for females or for COPD, had they existed. This is similar to the discriminatory power of many subchronic laboratory animal inhalation studies.

### 7.6. Conclusion, comparison of marketed cigarettes

With the most discriminatory non-clinical endpoints for the evaluation of mainstream smoke, such as chemical analysis or *in vitro* assays, differences in both directions can be detected between American-blend and Virginia-type market cigarettes. With the current state of knowledge, it is not clear which of these endpoints would be more relevant than others in predicting human health issues. No relevant difference between American-blend and Virginia-type cigarettes was detected for smoke exposure and effects, including measures for smoking dependence and smoking-related chronic diseases. Thus, with the current means of investigation of these endpoints, there is no indication of any relevant effect of using sugars as ingredient in commercial cigarettes.

## 8. Limitations and uncertainties of this assessment

The current assessment is considered a comprehensive evaluation of the evidence available on the potential effects of using sugars as ingredients. However, no such assessment can be without limitations, because cigarette smoke is a complex mixture of constituents and smoking-related diseases have complex chronic pathogeneses. Nevertheless, the scope of the current assessment went beyond standard ingredient assessments by industry or regulatory authorities, e.g. by modeling changes in smoke chemistry observed with research cigarettes into uptake distributions for smokers or by evaluating changes observed with research cigarettes in comparison to differences between principle types of market cigarettes, which included the possibility of indirectly assessing smoking behavior.

There are areas for which appropriate studies are missing and for practical reasons will continue to be missing. One example is the lack of a direct comparison of smokers smoking American-blend cigarettes with and without sugars applied as ingredients for the evaluation of a potential impact on smoking prevalence, smoke exposure, and smoking-related biological effects. An American-blend cigarette is defined by using sugars as ingredients, and the combustion products of these sugars in the tobacco matrix contribute to the overall flavor of such cigarettes thus enhancing the natural taste characteristics of the tobacco. A hypothetical clinical study comparing smoke exposure from cigarettes with and without sugars as ingredients would intrinsically be confounded by the uncommon taste of the test cigarettes without sugar. In order to assess this question in a more indirect manner, the best opportunity was considered to be a comparison of markets with smokers predominantly smoking American-blend cigarettes with added sugars and those predominantly smoking Virginia-type cigarettes without added sugars. However, these two types of cigarettes also differ in their natural content of sugars, resulting in similar or even lower overall sugar content in American-blend cigarettes compared to Virginia-type cigarettes.

There has been a broad agreement for the selection of smoke constituents or *in vitro* assays considered to be appropriate for such assessments between industrial and independent laboratories as well as the few emerging regulations (e.g. Health Canada, 2000; Gregg et al., 2004; Counts et al., 2005; Brazil National Health Monitoring Agency, 2007; Hyodo et al., 2007; Hammond and O'Connor, 2008; Moir et al., 2008; Gaworski et al., 2009), although most recently larger selections of smoke constituents have been suggested (Talhout et al., 2011; US Food and Drug Administration, 2011). The constituents considered most important by the World Health Organization Working Group on Tobacco Regulation (Burns et al., 2008; World Health Organization Study Group on Tobacco Product Regulation, 2008) were of course all included in the current assessment. Nevertheless, only a selection of constituents or toxicological activities is being assessed, assuming that the results of the selected endpoints would be representative of other constituents and biological endpoints. In order to increase the relevance of the non-clinical toxicological testing, subchronic inhalation studies have been included for the assessment of major cigarette ingredients, such as for sugars. A comprehensive array of potential target organs and tissues has often been included in the evaluation of these inhalation studies (in line with Organization for Economic Co-operation and Development, 2009) including, e.g. cardiovascular or reproductive organs, in order to minimize the probability of increasing the risks for the various smoking-related diseases and to avoid any new risks.

The non-clinical assay types reviewed here have been taken over from those used in other areas of regulatory toxicology. While their suitability for assessing the toxicity of smoke has not finally been established, this is not specifically the objective of cigarette ingredient testing anyhow. The objective is rather to determine that no significant toxicity is added to that intrinsic to tobacco smoke, either of the same type or new. Thus, classic regulatory toxicity tests may fit well to the testing of cigarette ingredients, if appropriately adapted to deal with the intrinsic toxicity of smoke and the resulting need for comparative testing. The bacterial mutagenicity assay may not be the optimal tool to evaluate the genotoxic property of smoke *per se*, but it may serve appropriately for the detecting of potential changes in genotoxic potency of smoke samples. For the testing of ingredients, the assay has been adapted to describe a linear dose-response relationship with smoke TPM, and the slope obtained with the test samples generated from experimental cigarettes with various ingredient application levels is compared in a quantitative manner (Roemer et al., 2002). The *in vitro* cytotoxicity assay has proven to be particularly useful for the assessment of cigarette smoke, as it relates to the irritative potency of smoke seen in *in vivo* studies. In *in vitro* genotoxicity studies with smoke, cytotoxicity has always been a limiting factor for dosing and interpretation thus demonstrating the relevance of this particular activity in assessing the complex toxicity of smoke. Finally, for the assessment of ingredients with relatively high application levels, subchronic inhalation studies have been used that have also been adapted to the comparative testing paradigm in terms of dosing and endpoint selection in order to study the relationship of the ingredient application level to the respective endpoints.

Uncertainties in the current assessment are also related to the limits in discriminatory power due to data variability in the studies underlying this assessment. The minimal detectable difference (as a measure for discriminatory power) in smoke chemistry, bacterial muta-genicity, and *in vitro* cytotoxicity has been determined to range between <10 and 30% of the mean responses assuming a significance level of 0.05 (a) and a type two error of 20% (β) (Roemer et al., 2010). Limitations in discriminatory power are particularly relevant when evaluating studies with a comparative design trying to detect differences against a control group with more or less the same qualitative chemical composition and a pronounced activity in the biological tests. In turn, this shows that the major contribution to toxicological potency, if not all, is indeed related to the intrinsic properties of cigarette smoke and not to the presence or absence of ingredients. Limitations in scope and discriminatory power and the uncertainty in interpreting effects seen in a particular study can at least be partially overcome by combining an array of assays with different but complementary information.

Finally, as with most comprehensive comparative assessments including a plethora of studies and endpoints, there are changes in individual endpoints pointing in both directions. There are endpoints, which change in a potentially favorable direction, e.g. the *in vitro* cytotoxicity of the particulate fraction or the yields of some N-nitrosamines, which have been implicated with smoking-related carcinogenicity. There are also changes in a potentially adverse direction, e.g. the yields of formaldehyde and benzene, which also have been implicated with smoking-related carcinogenicity.

Within the limitations of this assessment, the overall evidence does not suggest an increased risk or harm by using sugars as cigarette tobacco ingredients. Vice versa, these same limitations also hold true if one would consider omitting the use of sugars as ingredients in currently marketed cigarettes and replace it by tobacco instead. It cannot be excluded given the limited understanding of etiology and mechanisms, whether this replacement would even be triggering risks and harm towards an undesired direction. In a recent study, the reduction of sugar levels in tobacco by changing curing conditions and regulating the amount that is added was mentioned as a possibility to reduce the formation of carbonyl compounds (Talhout et al., 2011). These authors recognized, though, that any such action may result in shifting risks from one group of constituents to another, which would actually be the case based on the current assessment.

## 9. Overall conclusion

In a comprehensive assessment, the potential effects of using sugars as ingredients in American-blend cigarettes were evaluated. While some changes with sugar application were detected, the overall evaluation of all data considered on a weight-of-evidence basis suggests that the use of sugars would “add no significant toxicity to tobacco products and therefore could be considered safe in the context of this use” (stipulation taken from the US Institute of Medicine, 2001). This conclusion is based on the results of chemical analytical, *in vitro*, and subchronic inhalation studies with research cigarettes with and without sugars as tobacco ingredients. In the current assessment, the evaluation was extended beyond previous assessments to include information on smoke exposure and smoking behavior, which was achieved by comparing markets of predominantly American-blend or Virginia-type cigarettes. From these comparisons, e.g. for nicotine uptake levels, no indication of sugar application-related differences could be derived. The data analyzed do not support concerns that the use of sugars as ingredients would increase tobacco smoking dependence. No difference in mortality due to smoking-related diseases could be detected between American-blend and Virginia-type markets, which could have shed light on any population-based harm related to sugar application. In conclusion, thorough examination of the data available suggests that the use of sugars as ingredients in cigarette tobacco does not increase risk or harm beyond that inherent to cigarette smoking.
